# Electrochemically modified Corey–Fuchs reaction for the synthesis of arylalkynes. The case of 2-(2,2-dibromovinyl)naphthalene

**DOI:** 10.3762/bjoc.14.76

**Published:** 2018-04-23

**Authors:** Fabiana Pandolfi, Isabella Chiarotto, Marta Feroci

**Affiliations:** 1 Deptartment of Fundamental and Applied Sciences for Engineering (SBAI), Sapienza University of Rome, via Castro Laurenziano, 7, 00161, Rome, Italy

**Keywords:** arylalkyne, carbon–bromine bond cleavage, cathodic reduction, Corey–Fuchs reaction, 2-(2,2-dibromovinyl)naphthalene

## Abstract

The electrochemical reduction of 2-(2,2-dibromovinyl)naphthalene in a DMF solution (Pt cathode) yields selectively 2-ethynylnaphthalene or 2-(bromoethynyl)naphthalene in high yields, depending on the electrolysis conditions. In particular, by simply changing the working potential and the supporting electrolyte, the reaction can be directed towards the synthesis of the terminal alkyne (Et_4_NBF_4_) or the bromoalkyne (NaClO_4_). This study allowed to establish that 2-(bromoethynyl)naphthalene can be converted into 2-ethynylnaphthalene by cathodic reduction.

## Introduction

Terminal alkynes, due to the considerable triple-bond strength (839 kJ mol^−1^), are characterized by a moderate thermodynamic reactivity [1]. Nevertheless, both the C–C triple bond and the terminal C–H bond can be efficiently and selectively activated by metal or metal-free catalysts. Therefore, terminal alkynes can be considered as raw material (thus an important resource).

The use of terminal alkynes, activated by catalysts, as building blocks or intermediates in the synthesis of a large number of chemicals is extensively summarized in recent reviews [1–3]. The recently published papers confirm the present interest in the chemistry of terminal alkynes, e.g., in the synthesis of sulfinamides and isothiazoles [4], 1,3-enynes [5], α-monosubstituted propargylamines [6], 2-substituted pyrazolo[5,1-*a*]isoquinolines [7], etc.

**Scheme 1:**
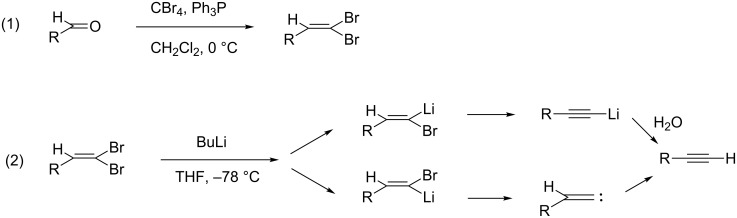
The Corey–Fuchs reaction.

Terminal alkynes can be prepared by dehydrohalogenation of vicinal dihalides or vinyl bromides using sodium in ammonia or strong bases [8]. Alternatively, the compounds are accessible by homologation of aldehydes following the Bestmann modification of the Seyferth–Gilbert reaction, using in situ generated dimethyl (diazomethyl)phosphonate [9–10]. Moreover, the aldehyde homologation to terminal alkynes can also be obtained using the Corey–Fuchs reaction [11]. This is a two-step reaction in which an aldehyde is at first converted into a 1,1-dibromoalkene with chain extension by one carbon atom through the reaction with carbon tetrabromide and triphenylphosphine (Scheme 1, reaction 1). The second step comprises the conversion of the 1,1-dibromoalkene into the corresponding alkyne by reaction with BuLi at −78 °C in THF (Scheme 1, reaction 2) [12].

Recently, a chemical modification of the second step of the Corey–Fuchs reaction was reported, in which the authors used Cs_2_CO_3_ as the base and performed the reaction in DMSO at 115 °C for 12 h [13]. Good to high yields of terminal alkynes were obtained (50–98%). Also DBU (4 equiv) in MeCN at room temperature is effective to carry out the second step of the Corey–Fuchs reaction, affording good to high yields of arylalkynes. In the latter reaction DBU acts both as base and as organocatalyst [14]. In all cases, an excess of a strong base or high temperature are necessary for the reaction to proceed. An overview on the importance of the Corey–Fuchs reaction for the synthesis of natural products has been pointed out by Heravi and co-workers recently [15].

As mentioned above the second step of the Corey–Fuchs reaction requires the cleavage of a C–Br bond. We thus envisaged if this could be achieved electrochemically via a selective cathodic cleavage of the C–Br bond. In this way, the reaction could be performed under mild conditions and in the absence of reducing agents or bases in the reaction mixture.

Electrochemical methods can be considered an environmentally friendly technique: they rely on the use of practically massless electrons (which are not converted to byproducts) instead of stoichiometric amounts of redox reagents and frequently these reactions are carried out at room temperature and at atmospheric pressure, etc. [16–19].

**Scheme 2:**
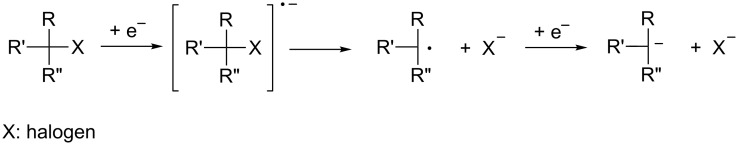
Electrochemical reduction of a carbon–halogen bond.

The electrochemical behavior of halogenated compounds has been extensively investigated [20–22]. The cleavage of the C–halogen bond, yielding (via a radical intermediate) the corresponding carbanion and halogen anion, can be achieved by a bielectronic cathodic process (Scheme 2). The electrolysis is carried out at a suitable controlled potential, i.e., at a potential that is negative enough to achieve the selective fission of the envisaged C–halogen bond [23].

Therefore, the reactive species is an electrochemically generated carbanion and the outcome of the reaction strongly depends on the complex reactivity of this intermediate. Moreover, this reactivity is influenced by the reaction conditions, such as the solvent, supporting electrolyte, electroinactive substrates, temperature, working potential and amount of consumed charge [24].

**Scheme 3:**
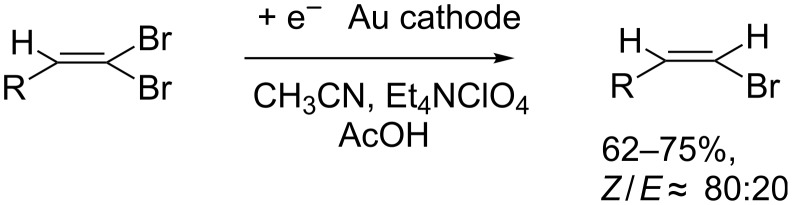
Electrochemical synthesis of vinyl bromides [25].

Our group intensively investigated the electrochemical behavior of 1,1-dibromoalkenes by means of cyclic voltammetry and electrolyses [25] and we reported the selective synthesis of vinyl bromides through the cathodic reduction of 1,1-dibromoalkenes in the presence of acetic acid. The electrolysis conditions in this transformation were optimized in order to avoid or minimize the formation of the terminal alkyne. The latter was obtained as the major product in the absence of a proton donor and its formation could be suppressed when performing the reaction with a Au cathode in acetonitrile (ACN) as the solvent and in the presence of an excess of acetic acid as the proton source. Under these conditions good yields of the vinyl bromides were obtained with preference of the Z-isomers (Scheme 3).

We have now reconsidered this investigation in order to obtain terminal alkynes and to avoid the formation of vinyl bromides. The scope of this paper is the determination of the electrolysis conditions for the transformation of 1,1-dibromoalkenes into the corresponding terminal alkynes, in order to carry out the second step of the Corey–Fuchs reaction under milder conditions.

**Scheme 4:**
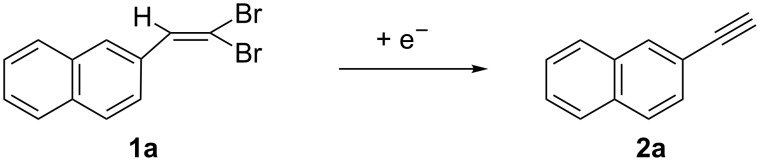
Scope of this work.

2-Ethynylnaphthalene (**2a**) is a small molecule with a high and selective biological activity. In particular, this molecule has been demonstrated to be a selective inactivator of cytochrome P-450 2B4 [26] and an inhibitor also of other cytochrome P-450 isoforms [27]. We thus decided to carry out our study using 2-(2,2-dibromovinyl)naphthalene (**1a**) as starting material for the synthesis of 2-ethynylnaphthalene (**2a,** Scheme 4).

## Results and Discussion

**Figure 1:**
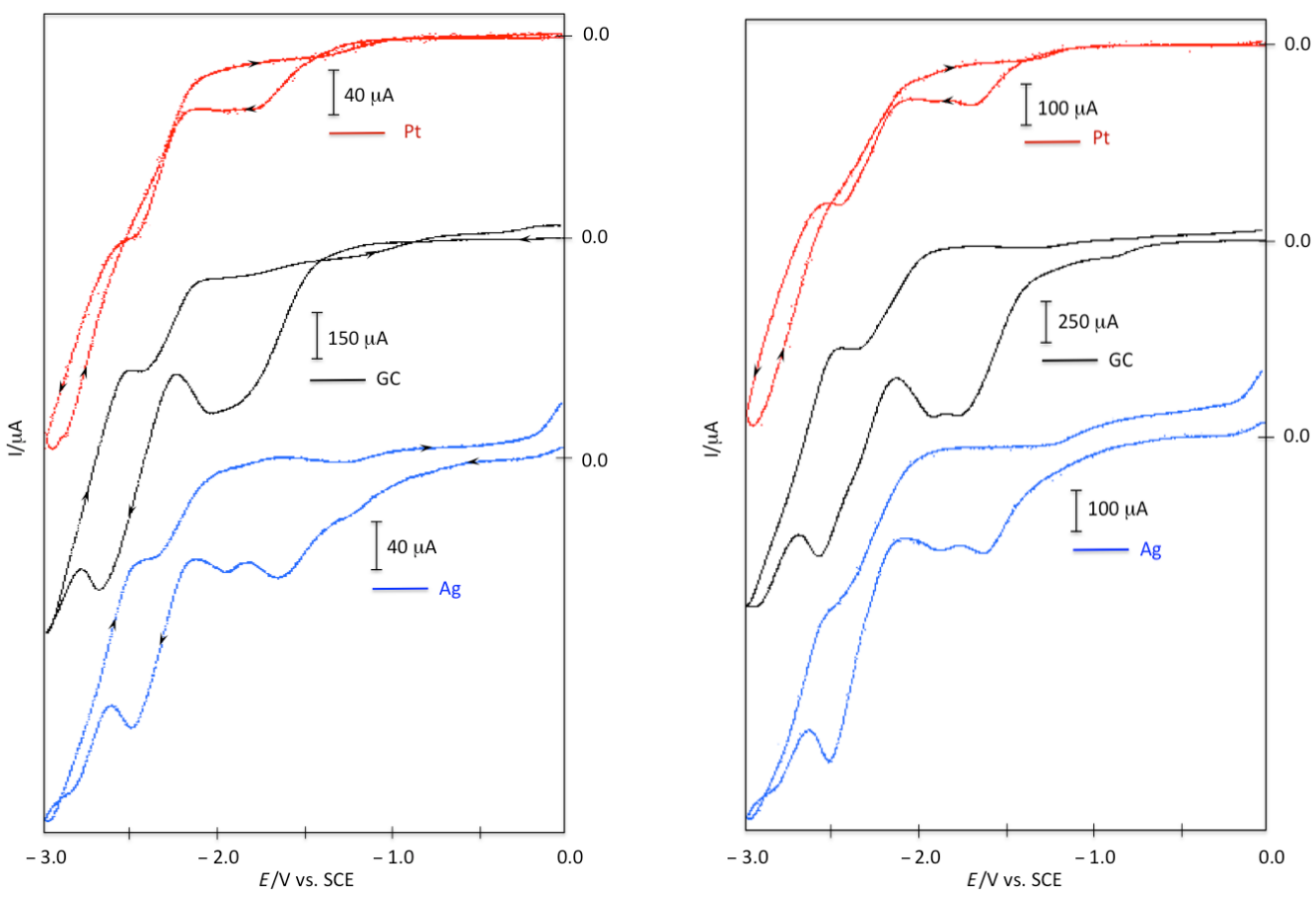
Voltammetric curves of **1a** 0.020 mol dm^−3^; Pt, glassy carbon (GC) or Ag cathode. ν = 0.2 V s^−1^, *T* = 25 °C; solvent left: DMF/Et_4_NBF_4_ 0.1 mol dm^−3^; right: ACN/Et_4_NBF_4_ 0.1 mol dm^−3^.

In our previous work [25], we found that the cathodic reduction of 2-(2,2-dibromovinyl)naphthalene (**1a**), carried out at the potential of the first voltammetric peak in ACN on a Au cathode and in the presence of an excess acetic acid, yielded the corresponding vinyl bromides (Scheme 3) in 75% yield (*Z/E* 82:18). The main product was 2-ethynylnaphthalene (**2a**, 65%) when the electrolysis was carried out in the absence of acetic acid as protonating agent (1.8 F consumed charge). Due to the importance of the latter product, we decided to reconsider this procedure in order to direct the synthesis towards the formation of the alkyne. We have therefore reconsidered the voltammetric behavior of **1a** at Pt, Ag and GC cathodes in DMF or ACN solutions (Figure 1).

The voltammetric curves of **1a** show the presence of different reduction peaks which are affected by the solvent and by the electrode material (see peak potential Table S1 in Supporting Information File 1). These voltammograms (and the data reported in Supporting Information File 1, Table S1) evidence the catalytic effect of the silver cathode in the C–Br bond reduction (*E*_p1_ is quite less negative on Ag cathode) [28–29], although this effect is more evident in DMF than in ACN. In any voltammogram, the cathodic peak at the less negative potential should be related to the cleavage of the C–Br bond. In order to confirm this statement, we carried out a first electrolysis in acetonitrile on a Pt cathode at the controlled potential of −1.75 V vs SCE, corresponding to the first reduction wave of **1a** (Table 1, entry 1). The current flow was stopped after the disappearance of **1a** (6.0 F). The only product was the expected alkyne **2a** (Scheme 4) with 69% yield. This result was in accordance with what reported in our previous work using a Au cathode (but with a much lower current efficiency – probably due to side reactions – when compared to the result obtained using a Au cathode in the previous paper) [25].

**Table 1:** Electrochemical synthesis of 2-ethynylnaphthalene (**2a**). Electrolysis conditions optimization (Scheme 5).^a^

entry	cathode	*E* or *I*^b^	F^c^	products (%)^d^			

				**2a**	**3a**	**4a**	**5a**
				
1^e^	Pt	−1.75 V	6.0	69	–	–	–
2	Pt	−2.00 V	3.0	80	–	traces	traces
3	Pt	−2.00 V	4.0	81	–	traces	–
4	Pt	−1.75 V	1.5	25	48	4	traces
5^f^	Pt	−1.75 V	2.3	27	–	–	58^g^
6	Pt	10 mA/cm^2^	3.0	46	–	41	–
7	Pt	5 mA/cm^2^	3.0	29	–	39	–
8	GC	−1.70 V	0.6	5	7	–	7
9	Ag	−1.80 V	3.0	72	–	6	–
10	Ag	−2.10 V	3.0	65	–	15	2
11^h^	Pt	−2.20 V	2.0	7	89	–	–
12^h^	Pt	−2.20 V	3.0	43	38	–	–

^a^Electrolysis conditions: divided cell, 5.0 mL of DMF (catholyte)/0.1 mol dm^−3^ Et_4_NBF_4_ containing **1a** (0.5 mmol), rt, N_2_ atmosphere. Anolyte: 2.0 mL same solvent. Working electrode: as in Table; anode: Pt; reference electrode: modified SCE (see Supporting Information File 1). The electrolyses were stopped after total consumption of starting **1a**. ^b^Controlled potential electrolyses: working potential *E* (Volts) reported vs SCE. Controlled current electrolyses: working current density *I* (mA/cm^2^) reported. ^c^Amount of charge: number of Faradays. ^d^Isolated yields, with respect to starting **1a**. ^e^ACN instead of DMF as solvent. ^f^3 Equivalents of acetic acid were present in the catholyte during electrolysis. ^g^Mixture of isomers: *Z*/*E* = 69:31. ^h^NaClO_4_ instead of Et_4_NBF_4_ as supporting electrolyte.

**Scheme 5:**
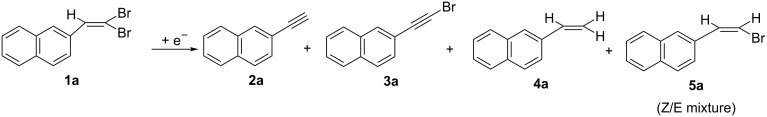
Possible products from the electrolysis of 2-(2,2-dibromovinyl)naphthalene (**1a**).

In order to ascertain the role of the solvent in this electrosynthesis, we carried out an electrolysis in DMF instead of ACN on a Pt cathode at the controlled potential of −2.00 V vs SCE, corresponding to the first reduction wave of **1a** (Table 1, entry 2). The current flow was stopped after the disappearance of **1a** (3.0 F). Also in this case the only product was the expected alkyne **2a** with a higher yield (80%).

An increase in the charge did not lead to an increase of the yield (81%, Table 1, entry 3). When the working potential was increased to −1.75 V and the electrolysis was stopped after the total consumption of **1a** (1.5 F), a mixture of products was obtained (Scheme 5 and Table 1, entry 4). In particular, a large amount (48%) of the brominated alkyne **3a** was isolated, along with traces of hydrogenated alkene **4a**. In order to confirm the effect of the presence of a proton donor, acetic acid was added to the solution and the electrolysis was carried out at the first cathodic peak potential (Table 1, entry 5). After 2.3 F (total consumption of starting material), the alkyne **2a** was isolated in 27% yield, while the major product was bromoalkene **5a** (mixture of *Z* and *E* isomers) in 58% yield. This result is very similar to what we reported in our previous work [25].

Also the electrochemical methodology has a dramatic effect on the products of the cathodic reduction of **1a**. In fact, carrying out the electrolysis under controlled current conditions (Table 1, entry 6) equimolar amounts of desired alkyne **2a** and of vinyl derivative **4a** (Scheme 5) were obtained when a current density of 10 mA/cm^2^ was used, while lowering the current density to 5 mA/cm^2^ did not alter significantly the reaction outcome (Table 1, entry 6 vs 7).

It is well known that the electrode material could influence the outcome of an electrosynthesis, so we carried out electrolyses of **1a** using a glassy carbon cathode (Table 1, entry 8) and a silver cathode (Table 1, entry 9). In both cases the working potential was that of the first reduction wave. In the case of glassy carbon, the electrolysis could not be terminated as the current flow stopped very early [30]. When a silver cathode was used, a good yield of desired alkyne **2a** was obtained (72%), along with a small amount of hydrogenated alkene **4a** (6%). In order to increase the yield of alkyne **2a** (and as **2a** reduction potential is much more negative, vide infra), we carried out a cathodic reduction of **1a** on a silver cathode at the second reduction wave potential (Table 1, entry 10). In this last case, the selectivity of the reaction dropped and a notable amount of hydrogenated alkene **4a** was obtained (15%), along with a lower yield of alkyne **2a** (65%).

The effect of a different supporting electrolyte was evaluated by substitution of Et_4_NBF_4_ with NaClO_4_. Also in this case the electrolysis was stopped after the complete consumption of starting **1a** (Table 1, entry 11). The change in supporting electrolyte led to a complete change in products. In fact, a very high yield of 2-(bromoethynyl)naphthalene (**3a**) was obtained (89%), along with only 7% of 2-ethynylnaphthalene (**2a**) after 2.0 F. Increasing the consumed charge to 3.0 F under the same experimental conditions, an equimolar mixture of bromoalkyne **3a** and alkyne **2a** was obtained, confirming the possibility of obtaining **2a** by cathodic reduction of **3a** (Table 1, entry 11 vs 12).

In order to better understand the electrochemical behavior of dibromoalkene **1a**, we carried out the voltammetric analysis of all isolated products (see Supporting Information File 1). The first cathodic peak potential of 2-(bromoethynyl)naphthalene (**3a**, Scheme 5) is very close to the first cathodic peak potential of 2-(2,2-dibromovinyl)naphthalene (**1a**), irrespective of the solvent and working electrode material. This renders impossible a selective cathodic reduction of **1a** in the presence of **3a**. The voltammetric behavior of 2-ethynylnaphthalene (**2a**) shows only one reduction peak at a potential that is quite more negative than the first cathodic peak of **1a** and **3a**, respectively, and corresponding to the third reduction peak of **1a** and to the second of **3a**. Also in this case the potential value is independent of the solvent and working electrode material. This voltammetric analysis shows that the cathodic reduction of both **1a** and **3a** could lead to the formation of the desired alkyne **2a**.

**Figure 2:**
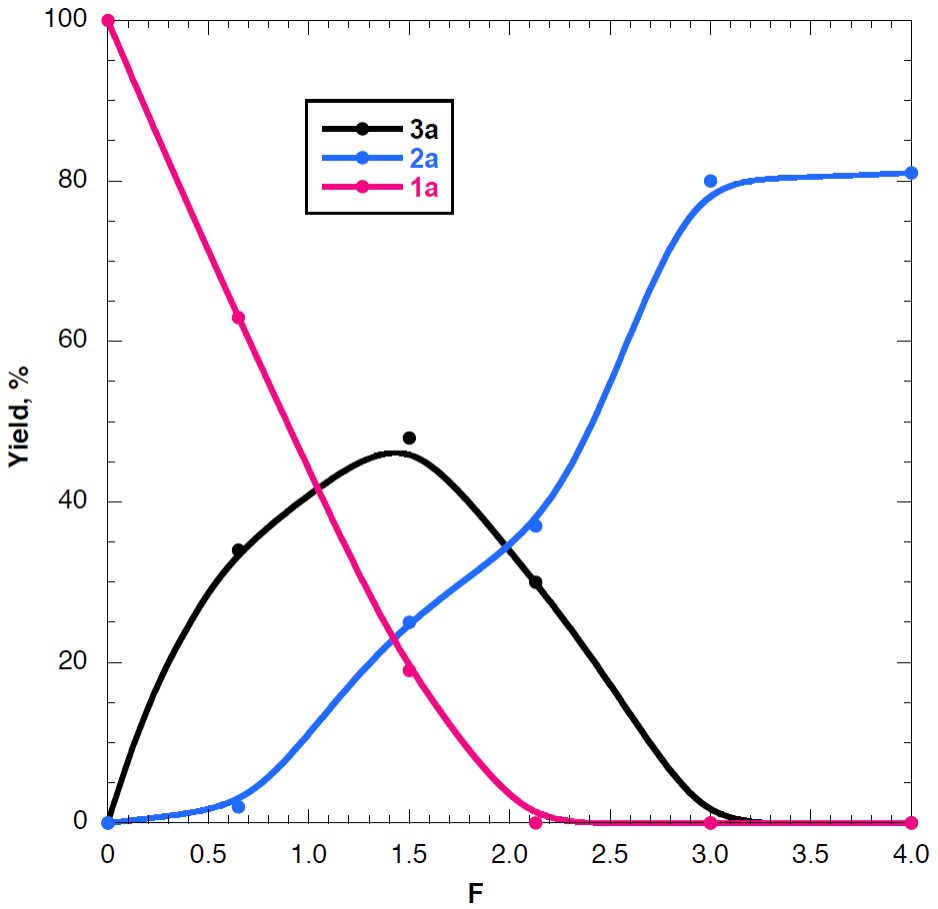
Variation of the amounts of **1a**, **2a**, and **3a** with the number of Faradays of **1a**.

To ascertain this hypothesis and to get information on the nature of the intermediates of the electrochemical process, we carried out the electrosynthesis under the optimized experimental conditions reported in Table 1, entry 2, analyzing the catholyte during the electrolysis. The yields of electrolysis products **2a** and **3a** were reported as a function of the number of Faraday (Figure 2).

The results of this last investigation (curves reported in Figure 2) show that i) the concentration of **1a** decreases and that of **2a** increases with increasing charge; ii) dibromoalkene **1a** is completely reduced after a consumption of 2.0 F, i.e., a value of charge near the theoretical value for the bielectronic reduction of a C–Br bond; iii) after a consumption of 2.0 F the yield of alkyne **2a** is 40% versus a yield of 80% after 3.0 F; iv) the analysis of the solution during the electrolysis shows the presence of bromoalkyne **3a**.

The concentration of **3a** initially increases and subsequently decreases upon increasing the charge; bromoalkyne **3a** is absent in the final solution. The maximum yield of **3a**, close to 50%, is reached after the consumption of about 1.5 F.

**Scheme 6:**
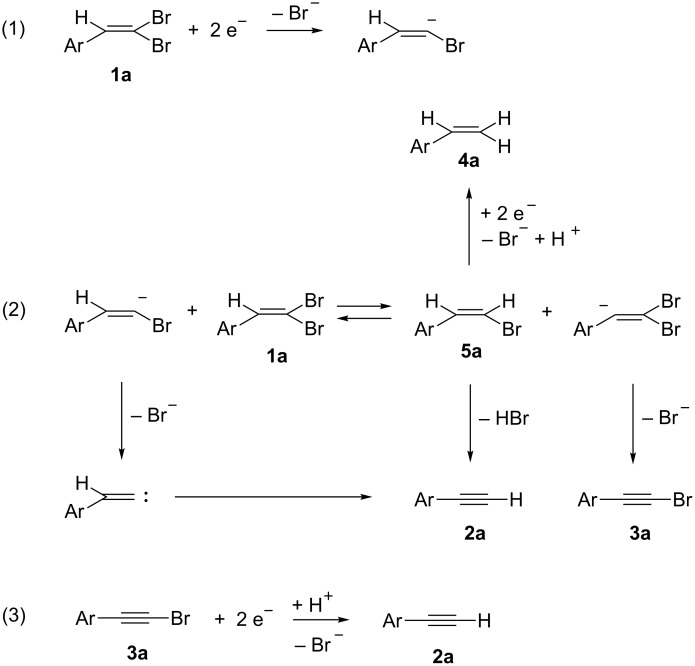
Mechanistic hypothesis for the synthesis of alkyne **2a** and bromoalkyne **3a** from 2-(2,2-dibromovinyl)naphthalene (**1a**). Alkene configurations are not defined.

Bromoalkyne **3a** and alkyne **2a** seem to be strictly related. In fact, the increase of **3a** corresponds to the decrease of starting **1a**, while the subsequent decrease of **3a** corresponds to the increase of **2a**. In addition the analysis of the electrolyzed solutions shows the presence of only a trace amount of vinyl bromide **5a**. Note that vinyl bromide **5a** is cathodically active at the working potential (see Supporting Information File 1). The overall analysis allows suggesting a mechanistic hypothesis (Scheme 6).

The bielectronic cathodic reduction of dibromoalkene **1a** leads to the cleavage of one C–Br bond and the formation of the corresponding vinyl anion (Scheme 6, reaction 1). An equilibrium of proton exchange between this electrogenerated carbanion and parent **1a** yields vinylbromide **5a** and a second vinyl anion (Scheme 2, reaction 2), which is converted to bromoalkyne **3a** by bromide elimination. Vinyl bromide **5a** can be cathodically reduced to 2-vinylnaphthalene (**4a**) or eliminate HBr to yield alkyne **2a**.

Bromoalkyne **3a** then can be reduced at the electrode to yield alkyne **2a**. The presence of a proton donor (Table 1, entry 5) increases the yield of **5a** and substitutes **1a** (as proton donor) in reaction 2 (Scheme 6).

The anion generated by cathodic reduction of dibromoalkene **1a** (Scheme 2, reaction 1) can also eliminate bromide (as reported in literature [31]), yielding the corresponding carbene (Scheme 2, reaction 2). This carbene can undergo a rearrangement to yield alkyne **2a**. According to the mechanism shown in Scheme 6, the formation of bromoalkyne **3a** competes with the formation of **2a** in reaction 2 and its rate of formation is comparable to that of **2a**. Since its reduction potential is close to that of **1a** (see Supporting Information File 1, Table S1 and Figure S2), it is further reduced to the alkyne **2a** (reaction 3 in Scheme 6) during the electrolysis.

**Scheme 7:**
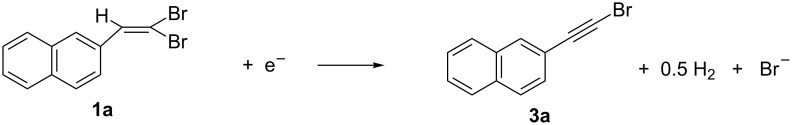
Possible reaction using NaClO_4_ as supporting electrolyte.

The various possible ways described in Scheme 6 are highly influenced by the reaction conditions. When the supporting electrolyte is NaClO_4_ instead of Et_4_NBF_4_, a different mechanism seems to be operative. In fact, following reactions (1) and (2) in Scheme 6, a maximum yield of 50% of **3a** can be obtained. It is thus possible that when using NaClO_4_ an electrogenerated base (OH^−^) is formed, due to the reduction of water and this base converts **1a** to **3a**. In fact, the Na^+^ cation is highly hydrophilic while the Et_4_N^+^ cation is hydrophobic. Thus, in DMF/NaClO_4_ the double layer would be constituted by the strongly solvated Na^+^(H_2_O)*_n_*, while in DMF/Et_4_NBF_4_, the double layer would be free of water. On Pt, a low hydrogen overvoltage material, it is then conceivable that the reduction of water to dihydrogen and hydroxide anions would be faster than the reduction of **1a**. The overall reaction would be a one-electron process catalyzed by water reduction (Scheme 7) [32].

It is thus possible by selecting the electrolysis conditions to synthesize selectively 2-ethynylnaphthalene (**2a**, Table 1, entry 2) or 2-(bromoethynyl)naphthalene (**3a**, Table 1, entry 11) in high yields.

**Scheme 8:**
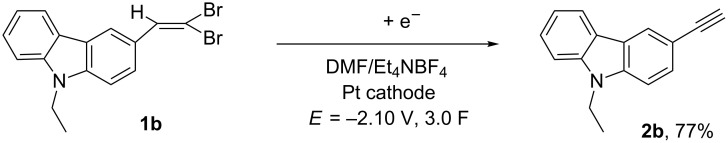
Electrochemical synthesis of 9-ethyl-3-ethynyl-9*H*-carbazole (**2b**).

Finally, to test the general applicability of the proposed electrochemical methodology, we submitted to electrolysis (under the optimized conditions reported in Table 1, entry 2), 3-(2,2-dibromovinyl)-9-ethyl-9*H*-carbazole (**1b**, Scheme 8). In fact, the corresponding alkyne **2b** is an important intermediate in the synthesis of molecules for organic electronics (e.g., organic light-emitting diodes [33] and organic field-effect transistors [34]). The voltammetric analysis showed a behavior similar to that of **1a** (see Supporting Information File 1) and thus the electrolysis was carried out at the second wave potential. 9-Ethyl-3-ethynyl-9*H*-carbazole (**2b**) was obtained in 77% yield.

**Scheme 9:**
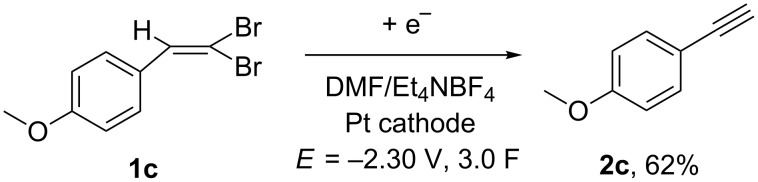
Electrochemical synthesis of 1-ethynyl-4-methoxybenzene (**2c**).

Similarly, when starting from 1-(2,2-dibromovinyl)-4-methoxybenzene (**1c**), the corresponding terminal alkyne **2c** was obtained in 62% yield (Scheme 9).

## Conclusion

The electrochemical methodology is shown to be a useful tool in organic synthesis. The possibility to direct the reaction towards different products simply by changing the electrolysis parameters (potential, solvent, supporting electrolyte, amount of charge, additives, etc.) and making use of electrons (as green, cheap, no byproduct-forming reagents) renders electrosynthesis attractive for organic chemists.

In particular, this work reported the selective synthesis of 2-ethynylnaphthalene or 2-(bromoethynyl)naphthalene in high yields by the cathodic reduction of 2-(2,2-dibromovinyl)naphthalene. The electrolyses were carried out in DMF solution (Pt cathode) under potentiostatic conditions; if the potential was fixed at −2.00 V (vs SCE) and the supporting electrolyte was Et_4_NBF_4_, and 2-ethynylnaphthalene was obtained in 80% yield after 3.0 F, while using NaClO_4_ as salt and a potential of −2.20 V 2-(bromoethynyl)naphthalene was obtained in 89% yield after 2.0 F. We also demonstrated that 2-(bromoethynyl)naphthalene can be cathodically converted to 2-ethynylnaphthalene. The extension of the method to two other substrates was successfully demonstrated. This methodology allows carrying out the second step of the Corey–Fuchs reaction under milder experimental conditions.

## Experimental

**Electrolyses**. Constant potential or current electrolyses were performed under a nitrogen atmosphere at 25 °C using an Amel 2053 potentiostat-galvanostat equipped with an Amel 731 integrator. All experiments were carried out in a divided glass cell separated through a porous glass plug filled with a layer of gel (i.e., methyl cellulose 0.5 vol % dissolved in DMF/Et_4_NBF_4_, 1.0 mol dm^−3^). Pt spirals (apparent area 0.8 cm^2^) were used as both cathode and anode, unless otherwise specified. Catholyte: 5 mL of DMF/0.1 M Et_4_NBF_4_; anolyte: 2 mL of the same solvent of catholyte. 2,2-Dibromovinylnaphthalene (0.5 mmol) was present in the catholyte during electrolysis. The number of Coulombs and the electrolysis potential/current were varied as reported in the text. At the end of the electrolysis, the catholyte was poured in an excess of water and extracted with petroleum ether 40–60 (3 × 20 mL). Flash column chromatography (eluent: petroleum ether/ethyl acetate from 100:0 to 90:10) gave purified products.

## Supporting Information

File 1 Detailed experimental procedures, NMR spectra and cyclic voltammetries.

## References

[1] Lei J, Su L, Zeng K, Chen T, Qiu R, Zhou Y, Au C-T, Yin S-F (2017). Chem Eng Sci.

[2] Chinchilla R, Nájera C (2014). Chem Rev.

[3] Ackermann L (2014). Acc Chem Res.

[4] Rodríguez M R, Beltrán Á, Mudarra Á L, Álvarez E, Maseras F, Díaz-Requejo M M, Pérez P J (2017). Angew Chem, Int Ed.

[5] Islas R E, Cárdenas J, Gaviño R, García-Ríos E, Lomas-Romero L, Morales-Serna J A (2017). RSC Adv.

[6] Takano S, Kochi T, Kakiuchi F (2017). Chem Lett.

[7] Liu H, Lu L, Hua R (2017). Tetrahedron.

[8] Campbell K N, Campbell B K (1963). Org. Synth..

[9] Müller S G, Liepold B, Roth G J, Bestmann H J (1996). Synlett.

[10] Roth G J, Liepold B, Müller S G, Bestmann H J (2004). Synthesis.

[11] Corey E J, Fuchs P L (1972). Tetrahedron Lett.

[12] Sahu B, Muruganantham R, Namboothiri I N N (2007). Eur J Org Chem.

[13] Zhao M, Kuang C, Yang Q, Cheng X (2011). Tetrahedron Lett.

[14] Morri A K, Thummala Y, Doddi V R (2015). Org Lett.

[15] Heravi M M, Asadi S, Nazari N, Lashkariani M B (2015). Curr Org Chem.

[16] Steckhan E, Arns T, Heineman W R, Hilt G, Hoormann D, Jörissen J, Kröner L, Lewall B, Pütter H (2001). Chemosphere.

[17] Frontana-Uribe B A, Little R D, Ibanez J G, Palma A, Vasquez-Medrano R (2010). Green Chem.

[18] Schäfer H J (2011). C R Chim.

[19] Horn E J, Rosen B R, Baran P S (2016). ACS Cent Sci.

[20] Casanova J, Reddy V P, Patai S, Rappoport Z (1995). Electrochemistry of the carbon−halogen bond. The Chemistry of Functional Groups, Supplement D2.

[21] Peters D G, Schäfer H J (2004). Oxidation and reduction of halogen-containing compounds. Encyclopedia of Electrochemistry.

[22] Torii S (2006). Electroreduction of Halogenated Compounds. Electroorganic Reduction Synthesis.

[23] Peters D G, Hammerich O, Speiser B (2016). Organic Electrochemistry.

[24] Martin E T, McGuire C M, Mubarak M S, Peters D G (2016). Chem Rev.

[25] Feroci M, Orsini M, Palombi L, Sotgiu G, Inesi A (2004). Electrochim Acta.

[26] Strobel S M, Szklark G D, He Y Q, Foroozesh M, Alworth W L, Roberts E S, Hollenberg P F, Halpert J R (1999). J Pharmacol Exp Ther.

[27] Beebe L E, Roberts E S, Fornwald L W, Hollenberg P F, Alworth W L (1996). Biochem Pharmacol.

[28] Mubarak M S, Peters D G (2017). Curr Opin Electrochem.

[29] Gennaro A, Isse A A, Mussini P R, Hammerich O, Speiser B (2016). Organic Electrochemistry.

[R30] 30The use of glassy carbon (GC) as cathode was not possible probably due to adsorption of material on the electrode surface, which led to electrical insulation.

[31] Abbas S, Hayes C J, Worden S (2000). Tetrahedron Lett.

[R32] 32We are grateful to a referee for suggesting this mechanistic hypothesis.

[33] Li Y-P, Fan X-X, Wu Y, Zeng X-C, Wang J-Y, Wei Q-H, Chen Z-N (2017). J Mater Chem C.

[34] Kato S-i, Noguchi H, Kobayashi A, Yoshihara T, Tobita S, Nakamura Y (2012). J Org Chem.

