# Supplementary material for: Electrochemically modified Corey–Fuchs reaction for the synthesis of arylalkynes. The case of 2-(2,2-dibromovinyl)naphthalene
Source: Beilstein J Org Chem. 2018 Apr 23;14:891–9. doi: 10.3762/bjoc.14.76 (PMC5942384; doi:10.3762/bjoc.14.76)
Supplement: File 1 — Detailed experimental procedures, NMR spectra and cyclic voltammetries. [file Beilstein_J_Org_Chem-14-891-s001.pdf]

Supporting Information

for

Electrochemically modified Corey–Fuchs reaction for the synthesis of arylalkynes. The case of 2-(2,2-dibromovinyl)naphthalene

Fabiana Pandolfi, Isabella Chiarotto and Marta Feroci*

Address: [1]Deptartment of Fundamental and Applied Sciences for Engineering (SBAI), Sapienza University of Rome, via Castro Laurenziano, 7, 00161, Rome, Italy.

Email: Marta Feroci - marta.feroci@uniroma1.it

*Corresponding author

Detailed experimental procedures, NMR spectra and cyclic voltammetries

Table of Contents

General remarks S2
Cyclic voltammetries S2
Electrolyses S2
Synthesis of 2,2-dibromoalkenes S3
Spectral data of electrolysis products S3
Peak potentials table for 2-(2,2-dibromovinyl)naphthalene and its electrolysis products S5
Voltammetric curves S6
^{1}H and ^{13}C NMR spectra S13

General remarks

Anhydrous *N,N*-dimethylformamide (DMF) and acetonitrile (ACN) were commercially available and used as received. Tetraethylammonium tetrafluoroborate was commercially available and kept under reduced pressure 12 h prior to use. Commercially available reagents were used without further purification. Flash column chromatography was carried out using Merck 60 silica gel (230–400 mesh) under pressure. GC–MS measurements were carried out on an SE 54 capillary column using a Hewlett-Packard 5890 series II gas chromatograph coupled with a Hewlett-Packard 5871 series II quadrupole mass selective detector. ESI mass spectra were recorded on a ThermoFinnigan LCQ Classic LC/MS/MS ion trap equipped with an ESI source and a syringe pump. Samples (10^{-5} M in MeOH/H_2O 80:20) were infused in the electrospray system at a flow rate of 5–10 µL min^{-1}. In order to promote the analyte ionization, 50 µL of 10^{-2} M HCOOH were added to the sample solutions. ^{1}H and ^{13}C NMR spectra were recorded at room temperature using a Bruker AC 200 spectrometer as $CDCl_3$ solution using the residual solvent signal as internal standard.

Cyclic voltammetry

Voltammetric measurements were performed using an Amel 2053 potentiostat-galvanostat equipped with an Amel 7800 interface in a three-electrode cell; acquisition software was a CorrWare for Windows version 2.8d1 Scribner, elaboration software was a CorrView for Windows version 2.8d1 Scribner.

492/PT/1, 492/GC/3 and 492/AG/1 Amel microelectrodes were employed, using a Pt wire counter electrode and modified saturated calomel electrode as reference electrode (mSCE: SCE with organic solvent junction; the oxidation peak potential, on GC electrode, of ferrocene in DMF/0.1 M Et_4NBF_4 with this reference electrode is E_{oxFc} = + 0.512 V.). The scan rate was ν = 0.2 V s^{-1}. All cyclic voltammetry measurements were recorded using 5 mL of DMF/0.1 M Et_4NBF_4 at 25 °C, unless otherwise specified.

Electrolyses

Constant potential or current electrolyses were performed under a nitrogen atmosphere, at 25 °C, using an Amel 2053 potentiostat-galvanostat equipped with an Amel 731 integrator. All the experiments were carried out in a divided glass cell separated through a porous glass plug filled with a layer of gel (i.e., methyl cellulose 0.5 vol % dissolved in DMF/Et_4NBF_4 1.0 mol dm^{-3}); Pt spirals (apparent area 0.8 cm^2) were used as both cathode and anode, unless otherwise specified. Catholyte: 5 mL of DMF/0.1 M Et_4NBF_4. Anolyte: 2 mL of the same solvent of catholyte; 0.5 mmol of starting 2,2-dibromovinylarene were present in the catholyte during electrolysis. The number of coulombs and the electrolysis potential/current were varied as reported in the text. At the end of the electrolysis, the catholyte was poured in an excess of water and extracted with petroleum ether 40–60 (3 × 20 mL). Flash column chromatography (eluent: petroleum ether/ethyl acetate from 100:0 to 90:10) gave purified products.

Synthesis of starting 2,2-dibromoalkenes

All starting 2,2-dibromoalkenes were synthesized following the procedure described in Uenishi, J.; Kawahama, R.; Yonemitsu, O.; Tsuji, J. *J. Org. Chem.* **1998**, *63*, 8965-8975. DOI: 10.1021/jo9812781.
All the compounds gave spectral data in accordance with the literature.

2-(2,2-Dibromovinyl)naphthalene (1a) (Rao, M. L. N.; Jadhav, D. N.; Dasgupta, P. *Org. Lett.* **2010**, *12*, 2048-2051)
R_f (petroleum ether 40-60) = 0.52. ^{1}H NMR ($CDCl_3$, 200 MHz): δ 8.03 (s, 1H), 7.88 -7.82 (m, 3H), 7.68-7.63 (m, 2H), 7.56-7.49 (m, 2H). ^{13}C NMR ($CDCl_3$, 50.3 MHz): δ 136.9, 133.0, 132.7, 128.3, 128.1, 128.0, 127.7, 126.7, 126.5, 125.6, 89.8 MS (EI), m/z (relative intensity): 314 (30%, $M^{+\cdot}$ + 4), 312 (65%, $M^{+\cdot}$ + 2), 310 (29%, $M^{+\cdot}$), 153 (15%), 152 (100%), 151 (29%).

3-(2,2-Dibromovinyl)-9-ethyl-9*H*-carbazole (1b) (Cheung, C. W.; Hu, X. *Chem. Eur. J.* **2015**, *21*, 18439–18444)
R_f (petroleum ether 40-60/ethyl acetate 90:10) = 0.60. ^{1}H NMR ($CDCl_3$, 200 MHz): δ 8.36 (s, 1H), 8.11 (d, *J* = 7.6 Hz, 1H), 7.71-7.67 (m, 2H), 7.54-7.23 (m, 4H), 4.38 (q, *J* = 7.2 Hz, 2H), 1.46 (t, *J* = 7.2 Hz, 3H). ^{13}C NMR ($CDCl_3$, 50.3 MHz): δ 140.3, 139.7, 137.5, 126.3, 126.1, 126.0, 122.8, 120.7, 120.5, 119.3, 108.7, 108.2, 86.5, 37.7, 13.8. MS (EI), m/z (relative intensity): 381 (33%, $M^{+\cdot}$ + 4), 379 (65%, $M^{+\cdot}$ + 2), 377 (35%, $M^{+\cdot}$), 366 (25%), 364 (54%), 362 (29%), 219 (19%), 204 (100%), 190(55%), 176 (30%).

1-(2,2-Dibromovinyl)-4-methoxybenzene (1c) (Rao, M. L. N.; Jadhav, D. N.; Dasgupta, P. *Org. Lett.* **2010**, *12*, 2048-2051)
R_f (petroleum ether 40-60/ethyl acetate 90:10) = 0.55. ^{1}H NMR ($CDCl_3$, 200 MHz): δ 7.52 (d, *J* = 8.6 Hz, 2H), 7.42 (s, 1H), 6.90 (d, *J* = 8.6 Hz, 2H), 3.84 (s, 3H). ^{13}C NMR ($CDCl_3$, 50.3 MHz): δ 159.7, 136.3, 129.9, 127.8, 113.8, 87.3, 55.3. MS (EI), m/z (relative intensity): 294 (49%, $M^{+\cdot}$ + 4), 292 (100%, $M^{+\cdot}$ + 2), 290 (53%, $M^{+\cdot}$), 279 (14%), 277 (29%), 275 (14%), 132 (60%), 89 (73%).

Electrolysis products

2-Ethynylnaphthalene (2a) (Pünner, F.; Hilt, G. *Chem. Commun.*, **2012**, *48*, 3617–3619)
R_f (petroleum ether 40-60) = 0.41. ^{1}H-NMR ($CDCl_3$, 200 MHz): δ 8.05 (s, 1H), 7.86 -7.78 (m, 3H), 7.56-7.49 (m, 3H), 3.16 (s, 1H). ^{13}C-NMR ($CDCl_3$, 50 MHz): δ 133.0, 132.8, 132.3, 128.5, 128.0, 127.8, 126.9, 126.6, 119.4, 84.0, 77.4. MS (EI), m/z (relative intensity): 153 (21%, $M^{+\cdot}$ + 1), 152 (100%, $M^{+\cdot}$), 151 (40%), 126 (9%), 76 (21%), 63 (17%).

2-(Bromoethynyl)naphthalene (3a) (Okutani, M. Mori Y. *J. Org. Chem.* **2009**, *74*, 442–444)
$\mathbf{R_f}$ (petroleum ether 40-60) = 0.51. ^{1}H-NMR ($CDCl_3$, 200 MHz): δ 7.99 (s, 1H), 7.84 -7.76 (m, 3H), 7.55-7.46 (m, 3H). ^{13}C-NMR ($CDCl_3$, 50 MHz): δ 132.9, 132.8, 132.1, 128.4, 128.0, 127.7, 127.7, 126.9, 126.6, 120.0, 80.4, 50.0. MS (EI), m/z (relative intensity): 232 (52%, $M^{+\cdot}$ + 2), 230 (50%, $M^{+\cdot}$), 151 (100%), 150 (70%), 74 (65%), 62 (51%).

9-Ethyl-3-ethynyl-9*H*-carbazole (2b) (Misra, R.; T. Jadhav, T.; Dhokale, B. ; P. Gautam, P.; Sharma, R.; Maragani, R.; Mobin, S. M. *Dalton Trans.*, **2014**, *43*, 13076–13086)
R_f (petroleum ether 40-60/ethyl acetate 90:10) = 0.55. ^{1}H NMR ($CDCl_3$, 200 MHz): δ 8.29 (s, 1H), 8.10 (d, *J* = 7.8 Hz, 1H), 7.65-7.25 (m, 5H), 4.36 (q, *J* = 7.2 Hz, 2H), 3.11 (s, 1H), 1.44 (t, *J* = 7.2 Hz, 3H). ^{13}C NMR ($CDCl_3$, 50.3 MHz): δ 140.3, 139.8, 129.6, 126.2, 124.7, 122.8, 122.4, 120.6, 119.4, 111.9, 108.7, 108.4, 85.1, 75.1, 37.6, 13.8. MS (ESI), m/z (M + H^+): 220.18.

1-Ethynyl-4-methoxybenzene (2c) (Rassadin, V. A.; Nicolas, E.; Six, Y. *Chem. Commun.*, **2014**, *50*, 7666-7669)
R_f (petroleum ether 40-60) = 0.50. ^{1}H-NMR ($CDCl_3$, 200 MHz): δ 7.44 (d, *J* = 7.6 Hz, 2H), 6.85 (d, *J* = 7.6 Hz, 2H), 3.82 (s, 3H), 3.01 (s, 1H). ^{13}C-NMR ($CDCl_3$, 50 MHz): δ 159.9, 133.6, 114.2, 113.9, 83.6, 55.2. MS (EI), m/z (relative intensity): 132 (32%, $M^{+\cdot}$), 117 (23%), 101 (15%), 89 (100%), 63 (81%), 62 (68%).

Tabe S1: Cathodic peak potentials from cyclic voltammetry experiments of 2-(2,2-dibromovinyl)naphthalene and its electrolysis products (2×10^{-2} M in DMF or CAN/0.1 M Et_4NBF_4). Working electrode: Pt, Ag, GC. $\nu = 0.2$ V s^{-1}, $T = 25$ °C. Reference electrode: SCE.

**Substrate**	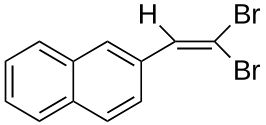 **1a**			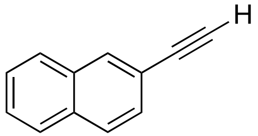 **2a**	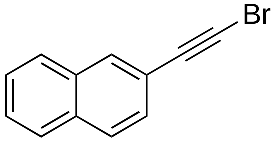 **3a**		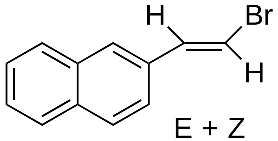 E + Z **5a**
**Electrode/Solvent**	$\mathbf{E_{p1}}$	$\mathbf{E_{p2}}$	$\mathbf{E_{p3}}$	$\mathbf{E_{p1}}$	$\mathbf{E_{p1}}$	$\mathbf{E_{p2}}$	$\mathbf{E_{p1}}$
Pt/ACN	-1.69 V	-1.91 V	-2.42 V	-2.36 V	-1.75 V	-2.32 V	
Pt/DMF	-1.79 V	-1.96 V	-2.47 V	-2.47 V	-	-	-2.09 V
GC/ACN	-1.74 V	-1.91 V	-2.57 V	-2.47 V	-1.74 V	-2.43 V	-1.92 V
GC/DMF	-1.83 V	-2.05 V	-2.67 V	-2.64 V	-1.77 V	-2.40 V	
Ag/DMF	-1.64 V	-1.94 V	-2.49 V	-2.42 V	-1.68 V	-2.34 V	
Ag/ACN	-1.62 V	-1.87 V	-2.52 V	-2.44 V	-1.59 V	-2.38 V	-2.00 V

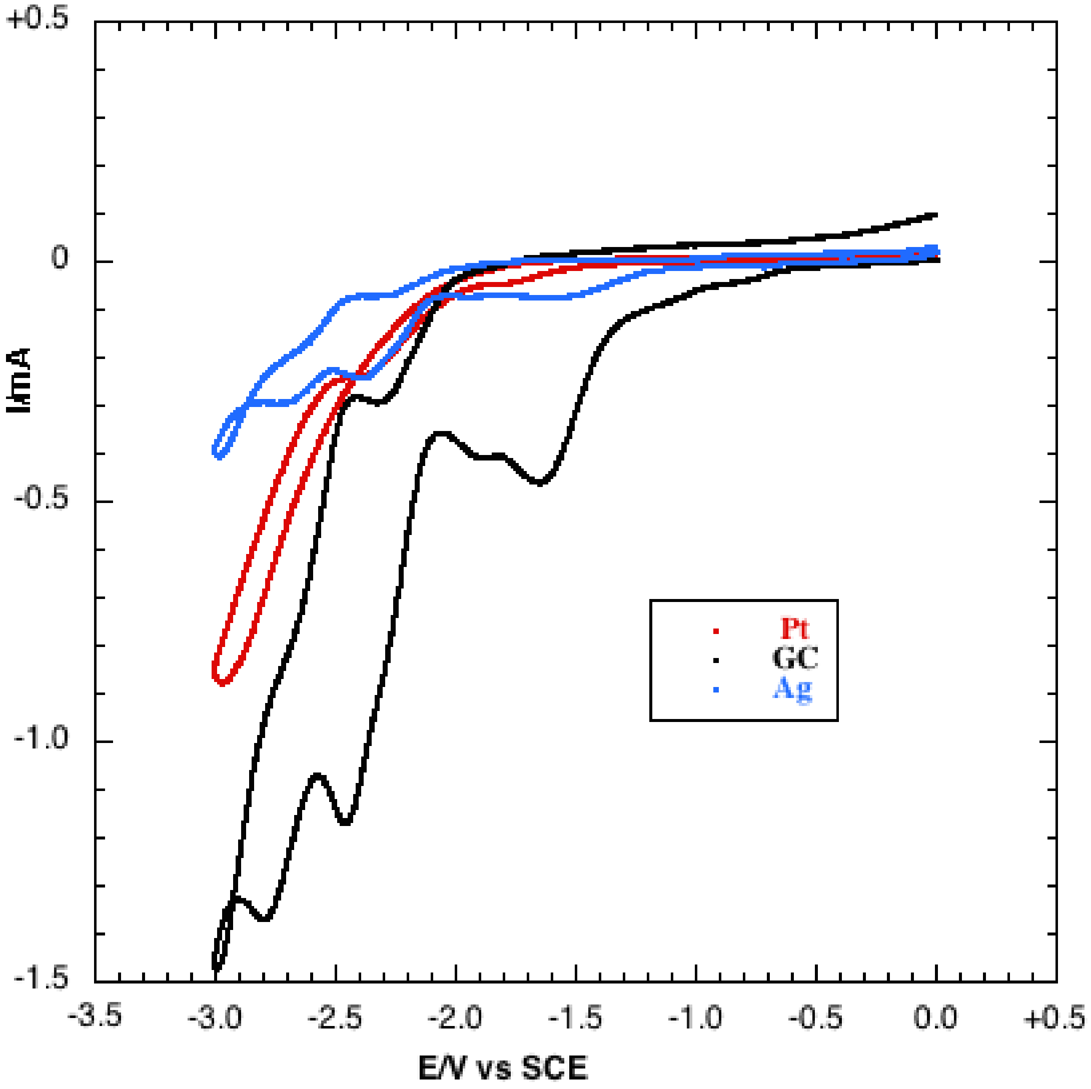

Figure S1: Cyclic voltammograms of 2-(bromoethynyl)naphthalene (**3a**), 2 × 10^{-2} M in CAN/0.1 M Et_4NBF_4. Working electrode: Pt, Ag, GC. ν = 0.2 V s^{-1}, T = 25 °C.

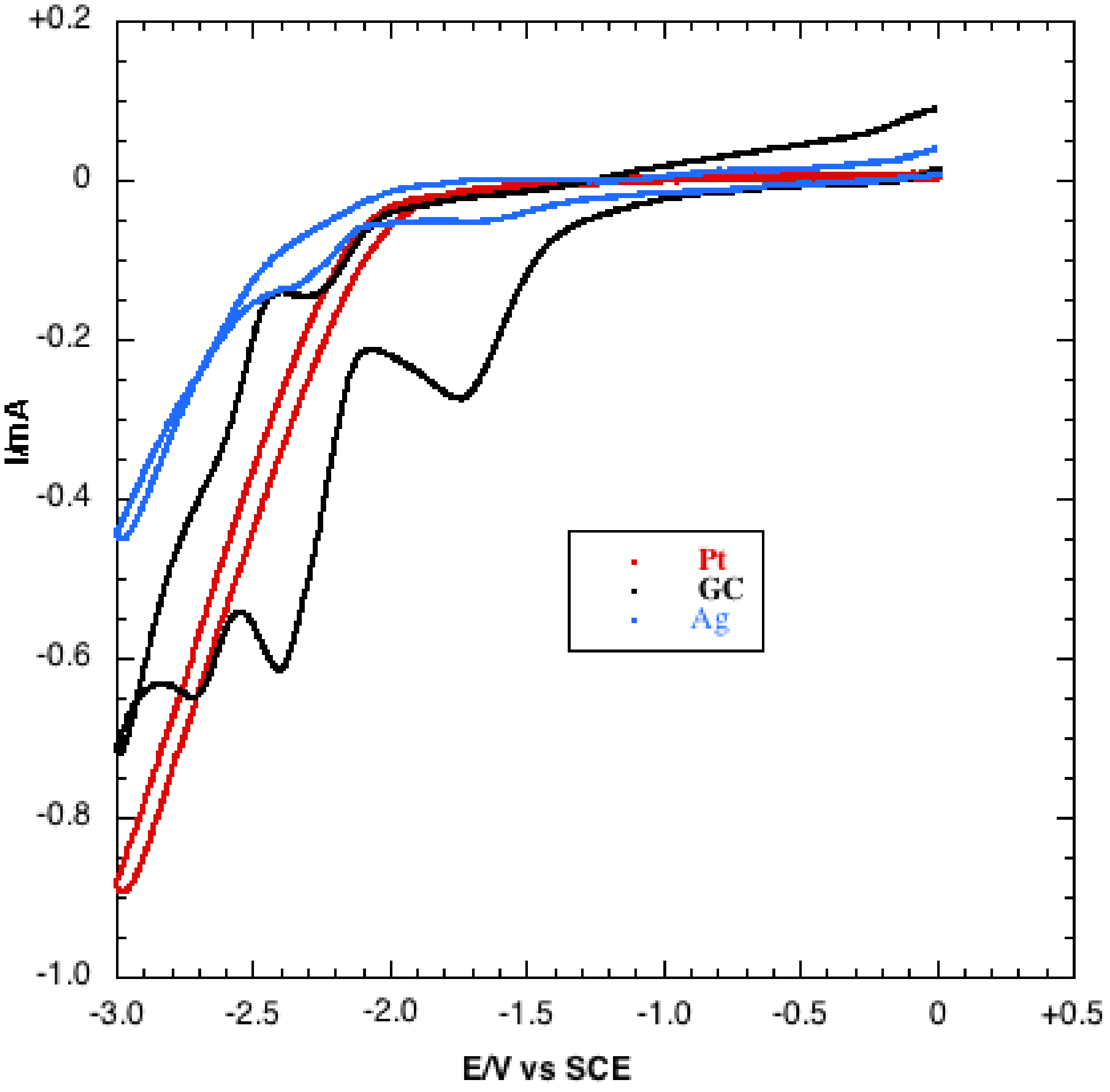

Figure S2: Cyclic voltammograms of 2-(bromoethynyl)naphthalene (**3a**), 6×10^{-3} M in DMF/0.1 M Et_4NBF_4. Working electrode: Pt, Ag, GC. $\nu = 0.2$ V s^{-1}, $T = 25$ °C.

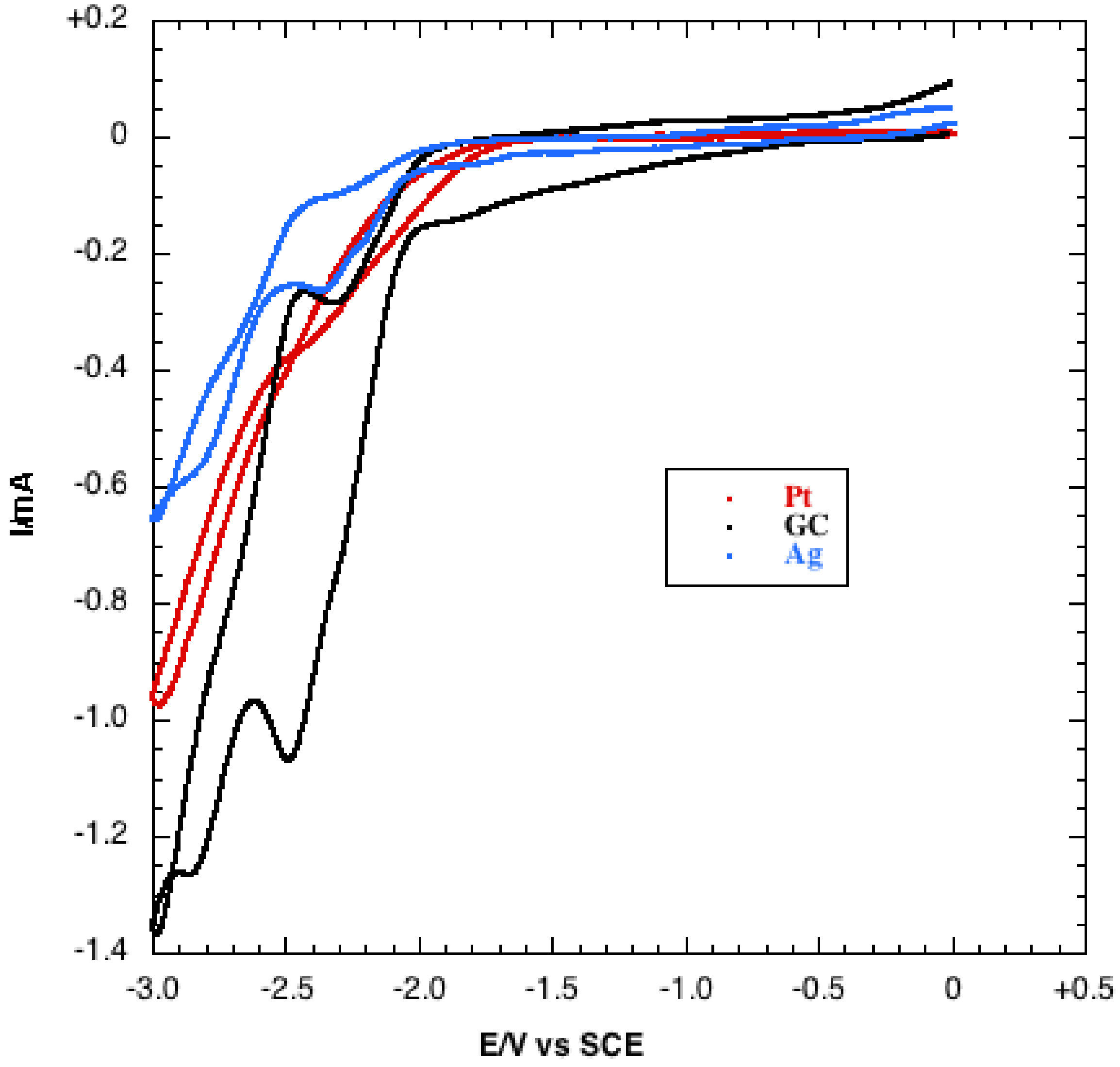

Figure S3: Cyclic voltammograms of 2-ethynylnaphthalene (**2a**), 2 × 10^{-2} M in CAN/0.1 M Et_4NBF_4. Working electrode: Pt, Ag, GC. ν = 0.2 V s^{-1}, T = 25 °C.

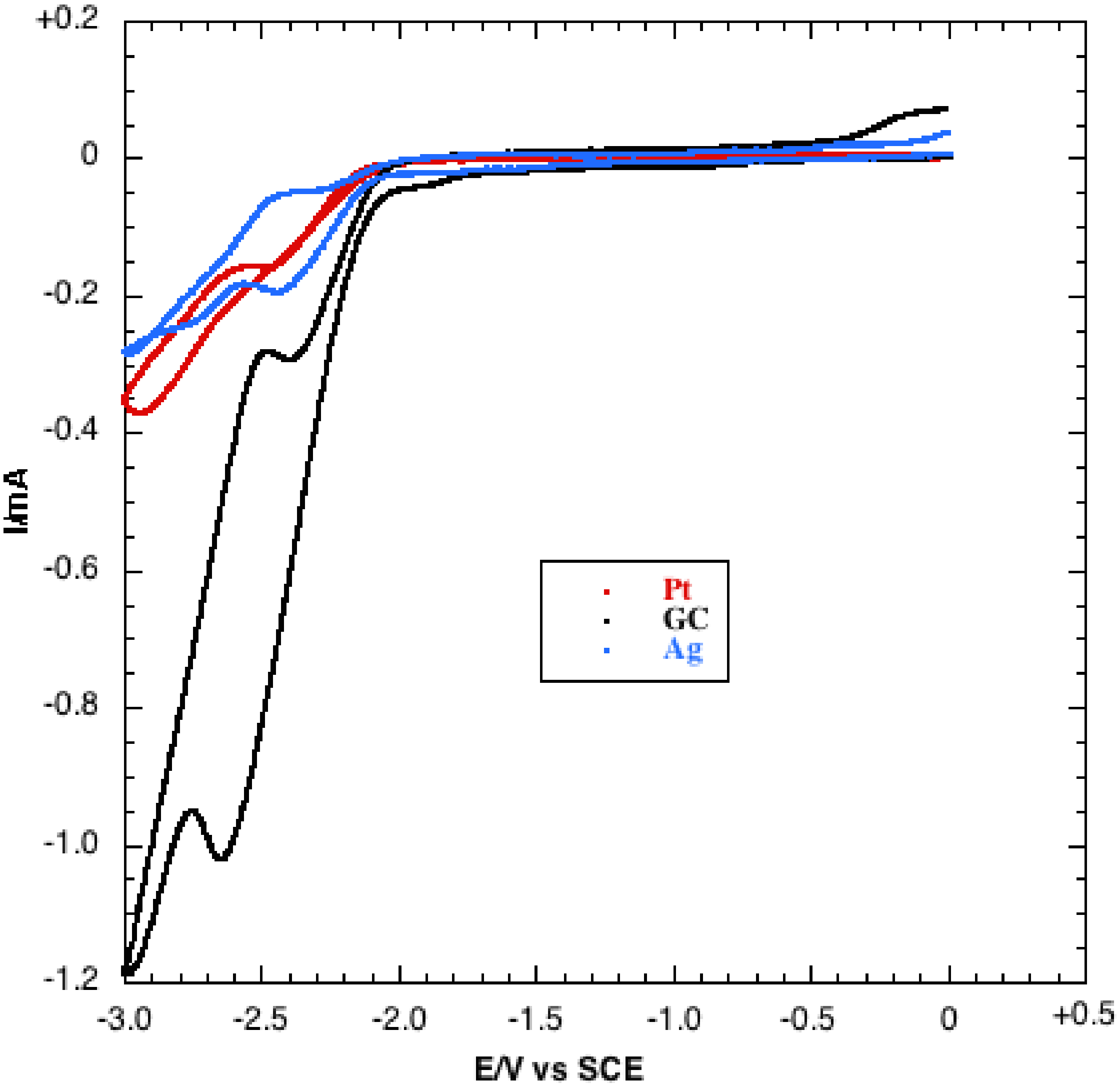

Figure S4: Cyclic voltammograms of 2-ethynylnaphthalene (**2a**), 2×10^{-2} M in DMF/0.1 M Et_4NBF_4. Working electrode: Pt, Ag, GC. $\nu = 0.2$ V s^{-1}, $T = 25$ °C.

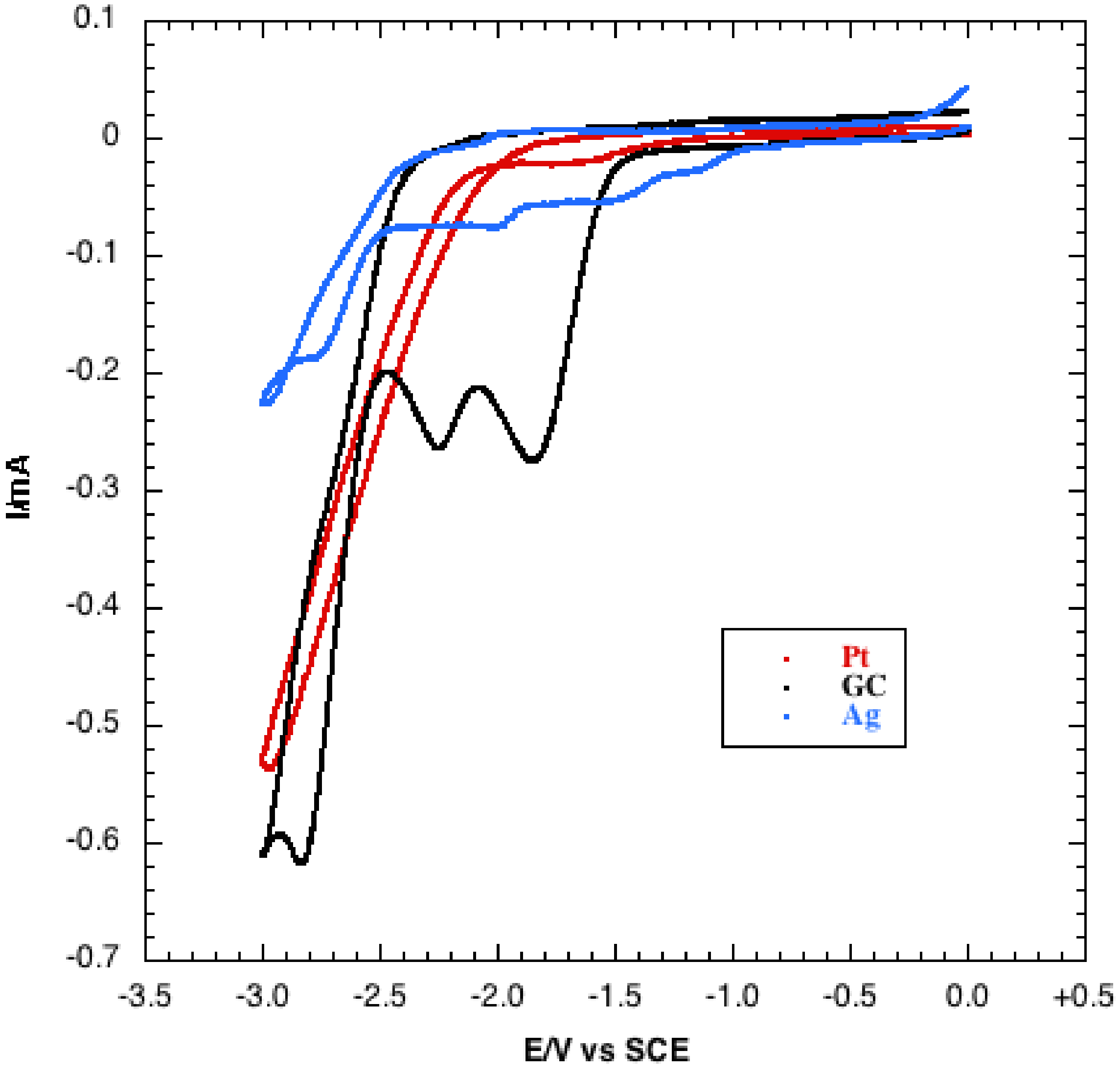

Figure S5: Cyclic voltammograms of 1-(2,2-dibromovinyl)-4-methoxybenzene (**1c**), 2 × 10^{-2} M in DMF/0.1 M Et_4NBF_4. Working electrode: Pt, Ag, GC. ν = 0.2 V s^{-1}, T = 25 °C.

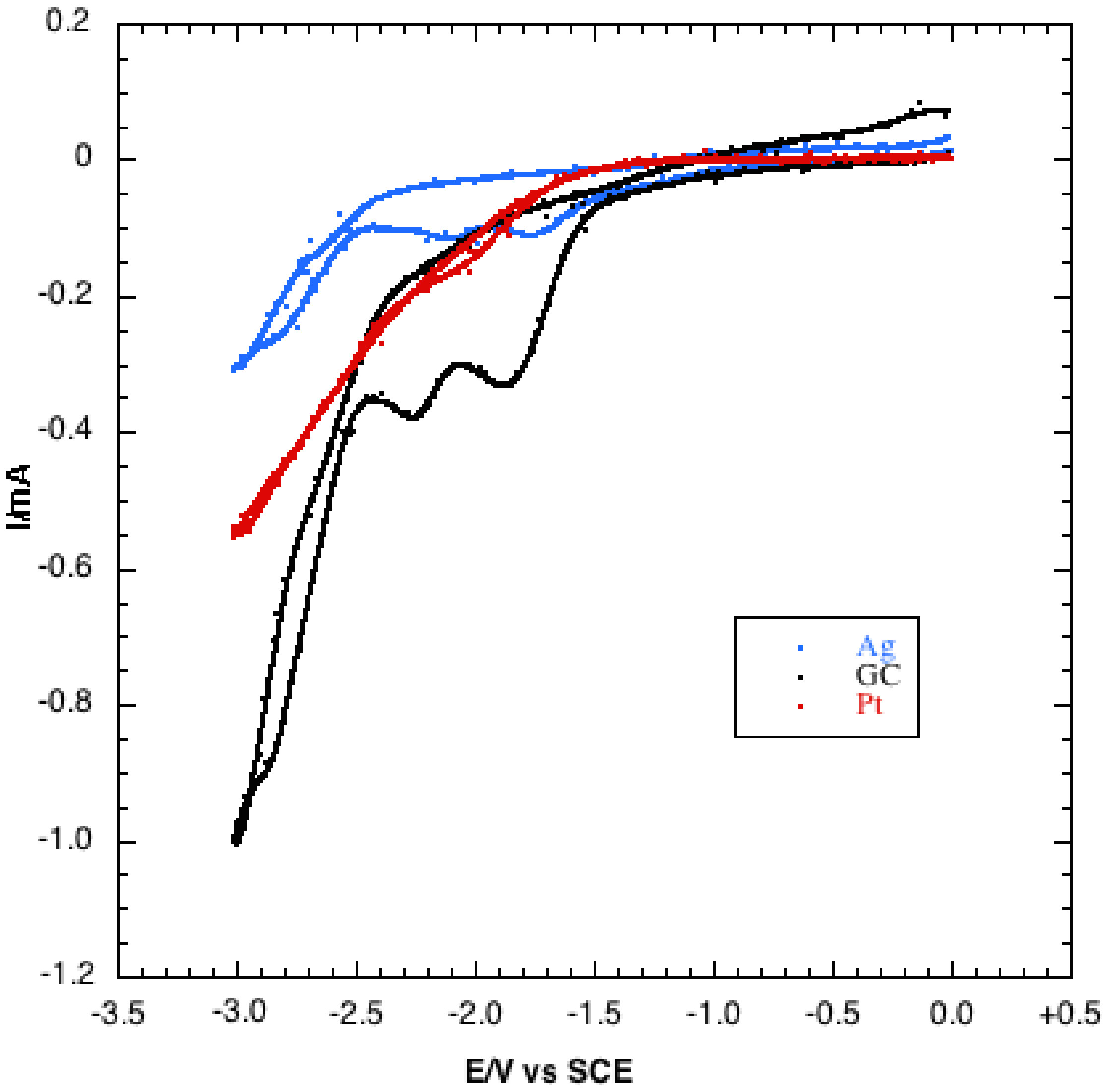

Figure S6: Cyclic voltammograms of 3-(2,2-dibromovinyl)-9-ethyl-9*H*-carbazole (**1b**), 2 × 10^{-2} M in DMF/0.1 M Et_4NBF_4. Working electrode: Pt, Ag, GC. ν = 0.2 V s^{-1}, T = 25 °C.

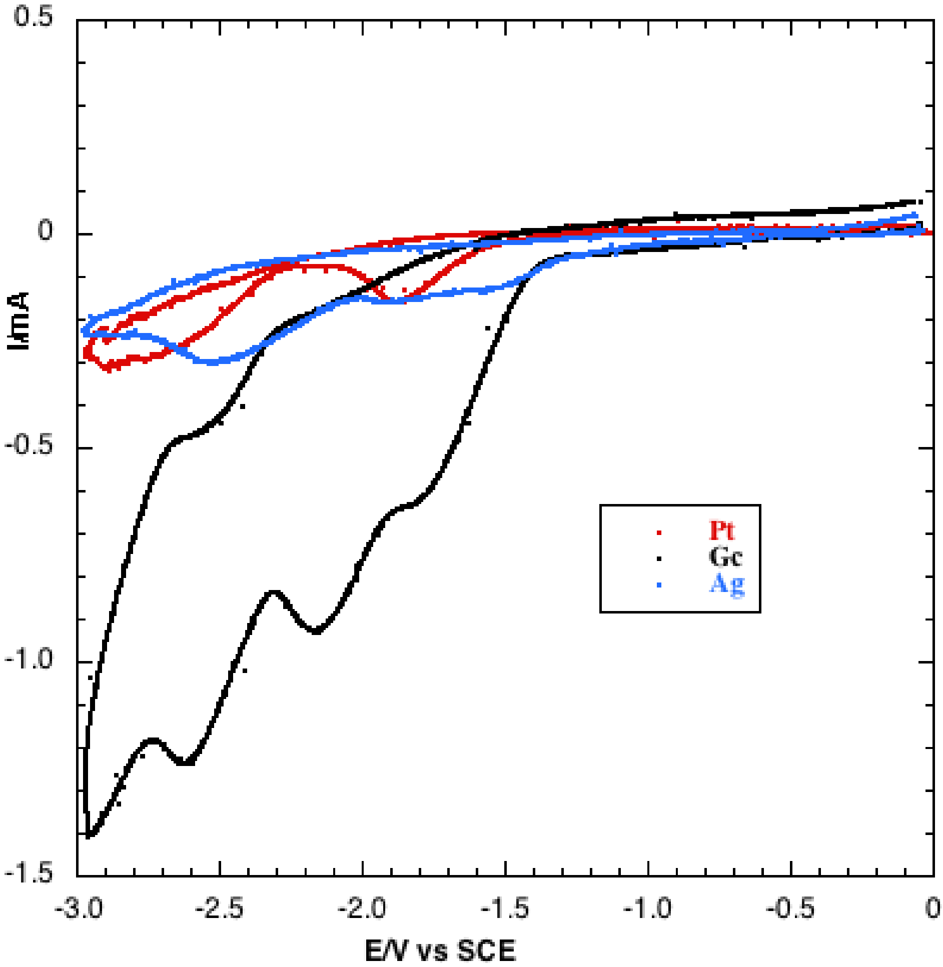

Figure S7: Cyclic voltammograms of 2-(2,2-dibromovinyl)naphthalene (**1a**), 2 × 10^{-2} M in DMF/0.1 M $NaClO_4$. Working electrode: Pt, Ag, GC. ν = 0.2 V s^{-1}, T = 25 °C.

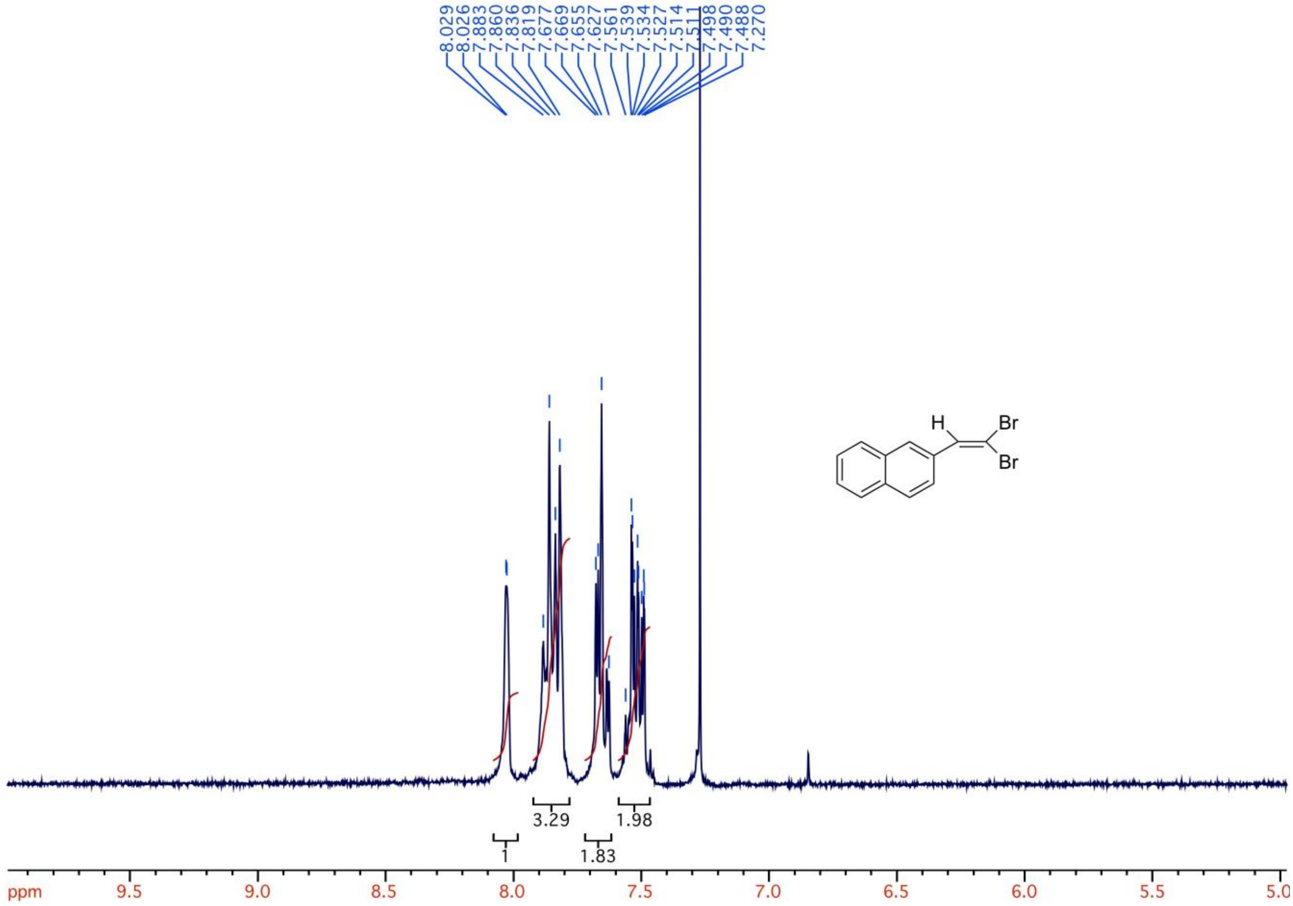

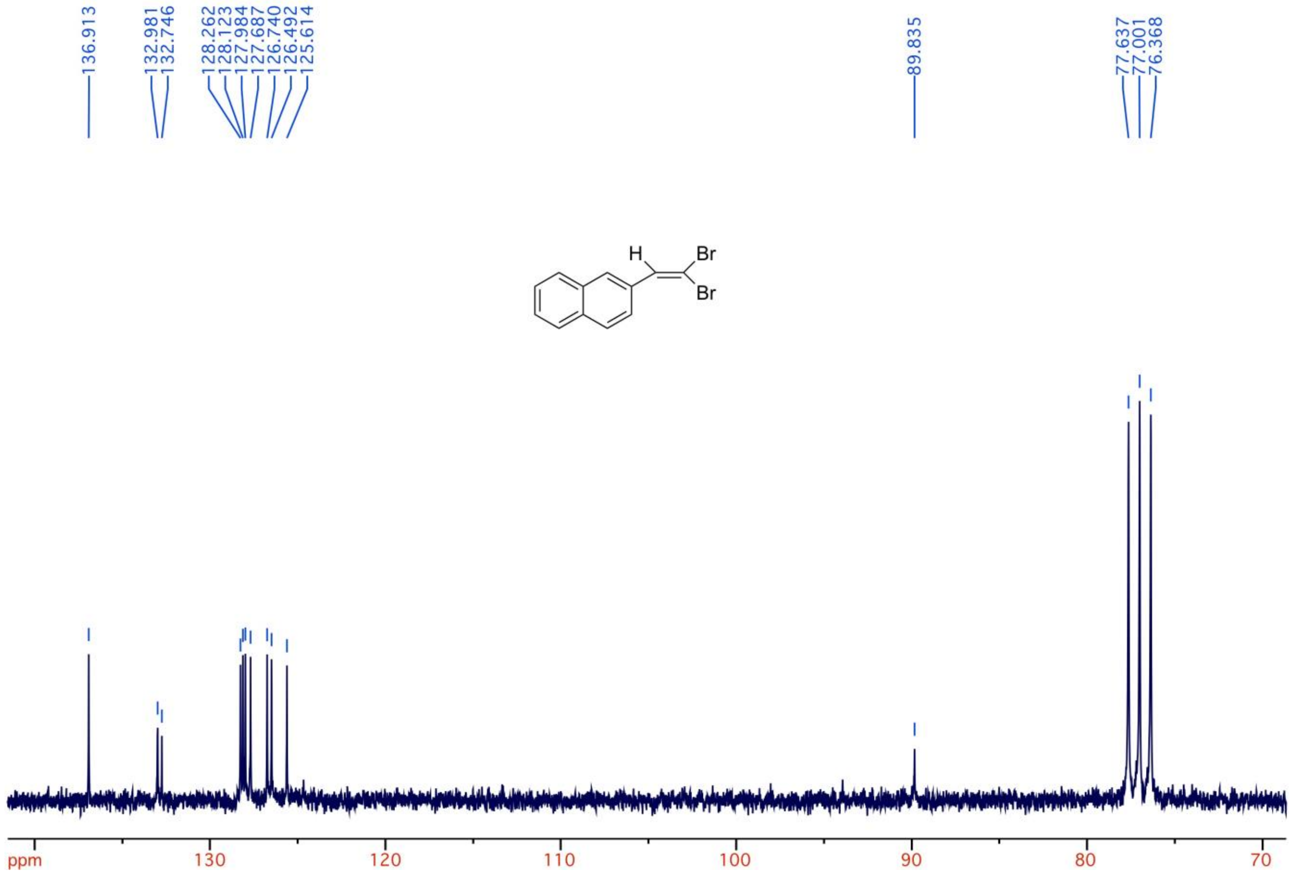

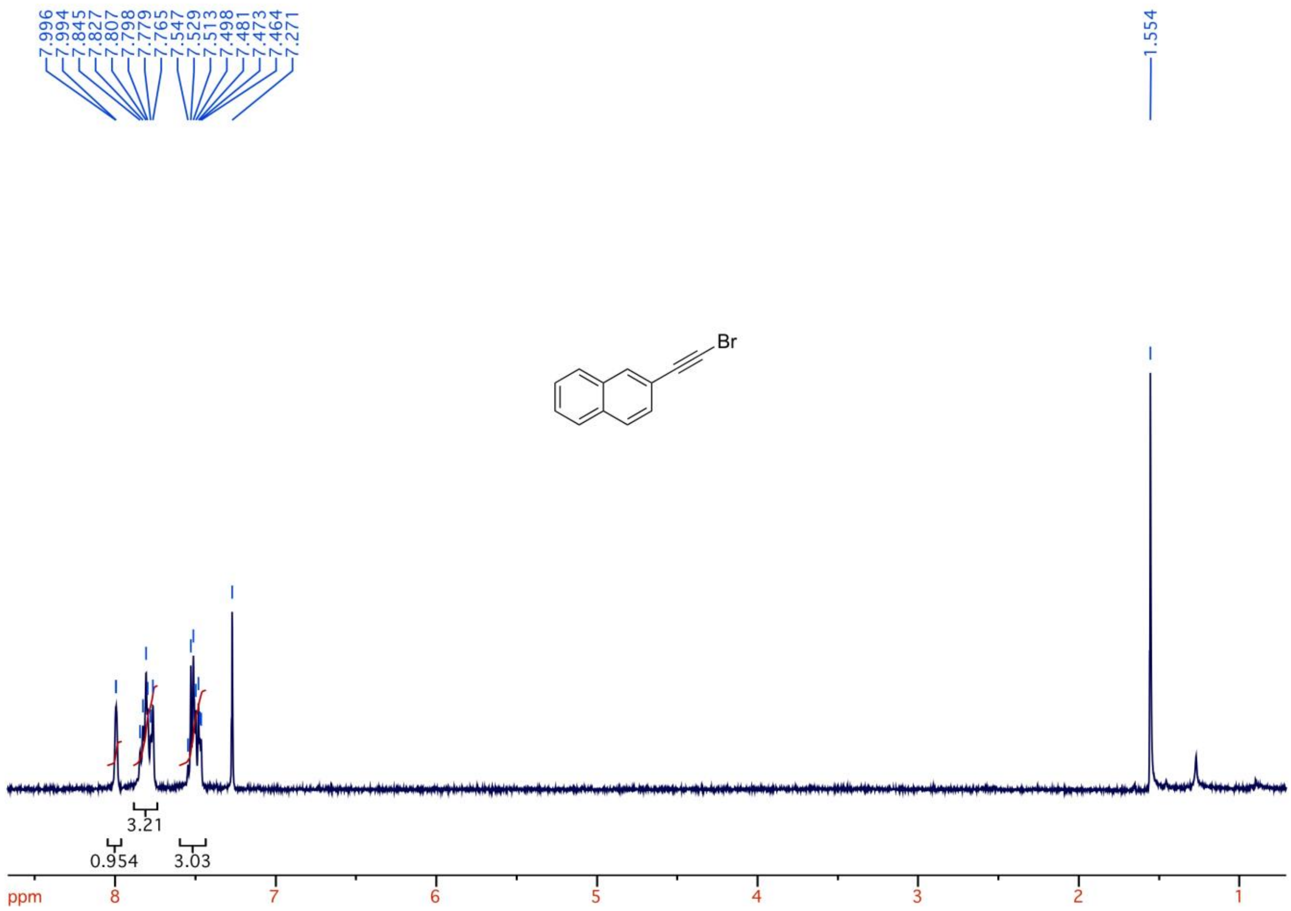
7.996
7.994
7.845
7.827
7.807
7.798
7.779
7.765
7.547
7.529
7.513
7.498
7.481
7.473
7.464
7.271
1.554
Br
3.21
0.954
3.03
ppm
8
7
6
5
4
3
2
1

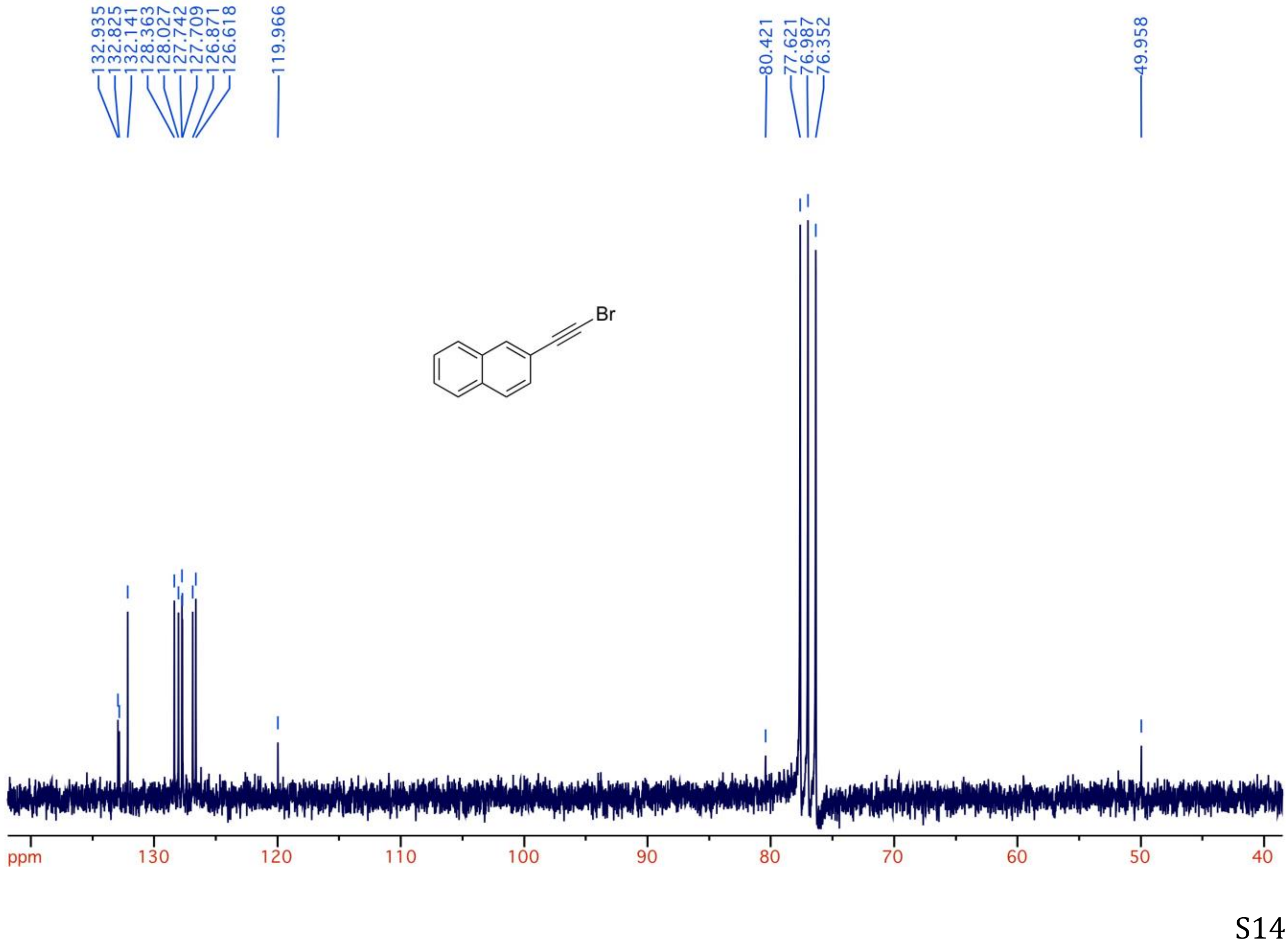
132.935
132.825
132.141
128.363
128.027
127.742
127.709
126.871
126.618
119.966
80.421
77.621
76.987
76.352
49.958
Br
ppm
130
120
110
100
90
80
70
60
50
40

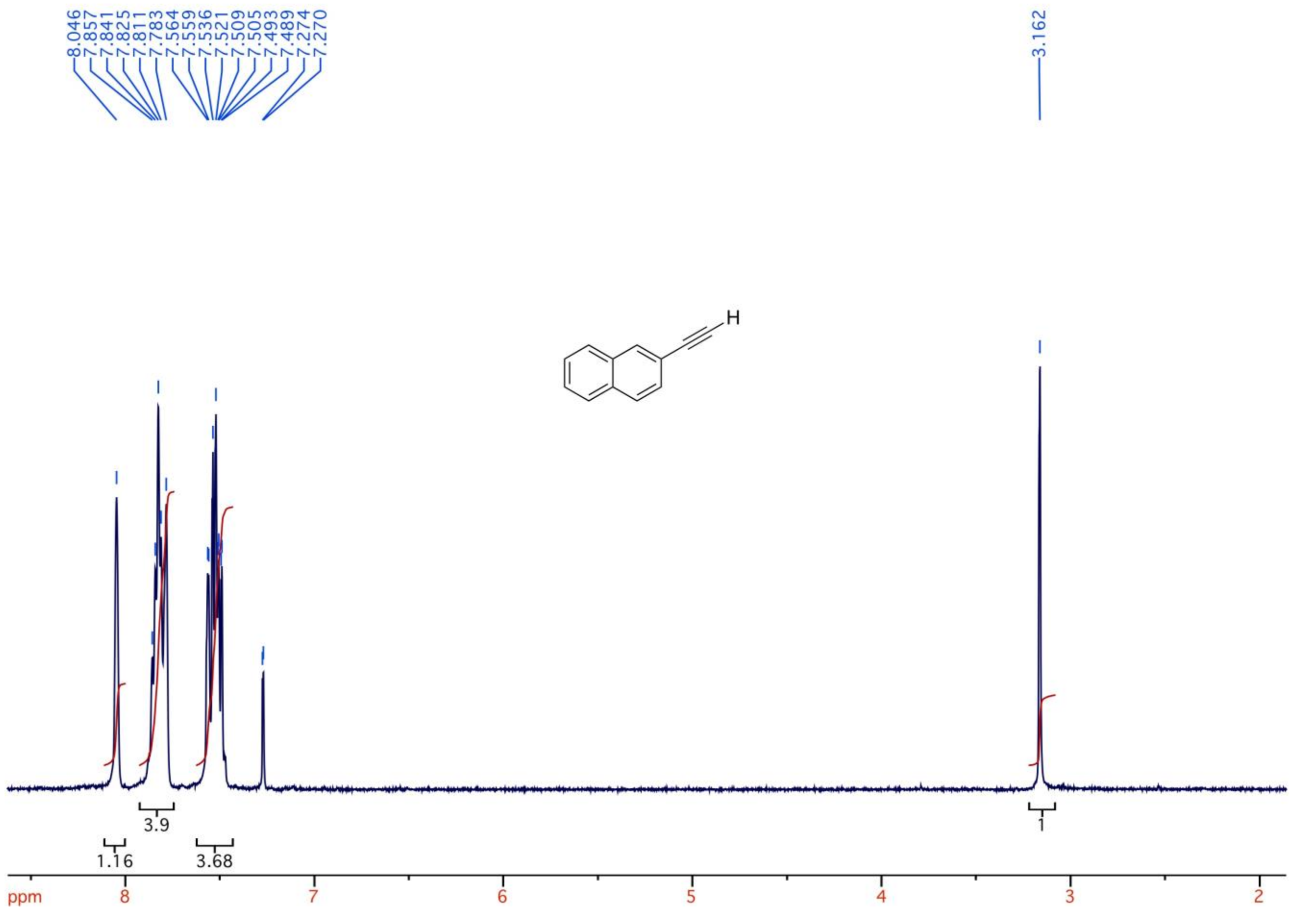
8.046
7.857
7.841
7.825
7.811
7.783
7.564
7.559
7.536
7.521
7.509
7.505
7.493
7.489
7.274
7.270
3.162
H
3.9
1.16
3.68
1
ppm
8
7
6
5
4
3
2

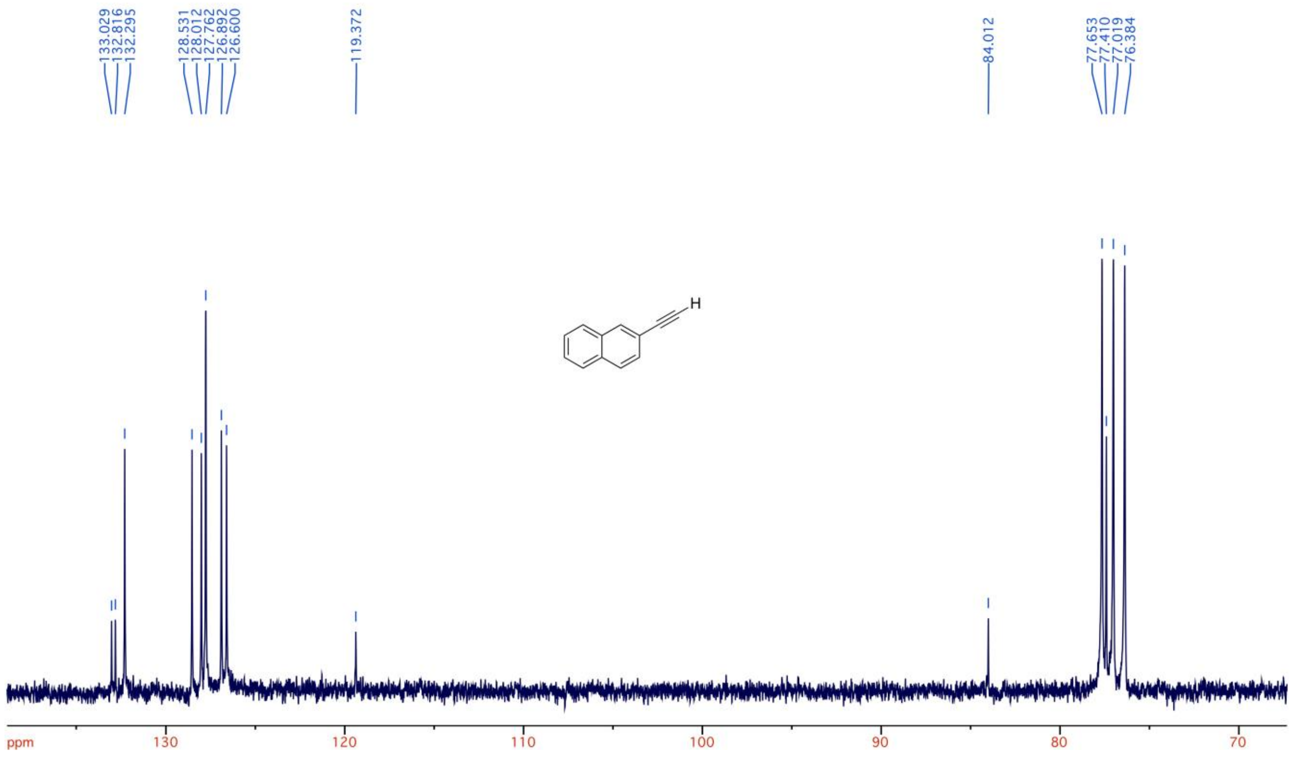
133.029
132.816
132.295
128.531
128.012
127.762
126.892
126.600
119.372
84.012
77.653
77.410
77.019
76.384
H
ppm
130
120
110
100
90
80
70

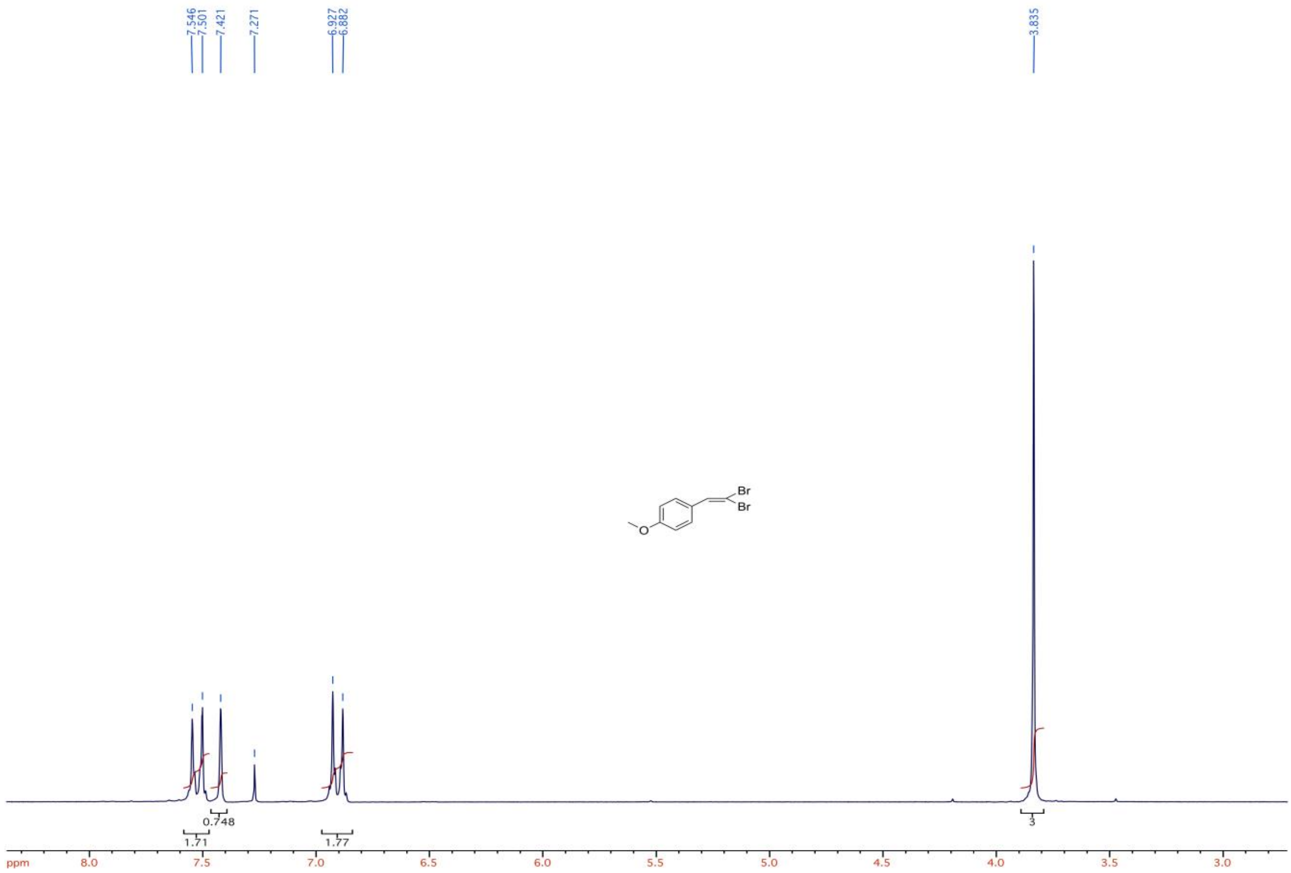

7.546
7.501
7.421
7.271
6.927
6.882
3.835
Br
Br
O
0.748
1.71
1.77
3
ppm
8.0
7.5
7.0
6.5
6.0
5.5
5.0
4.5
4.0
3.5
3.0

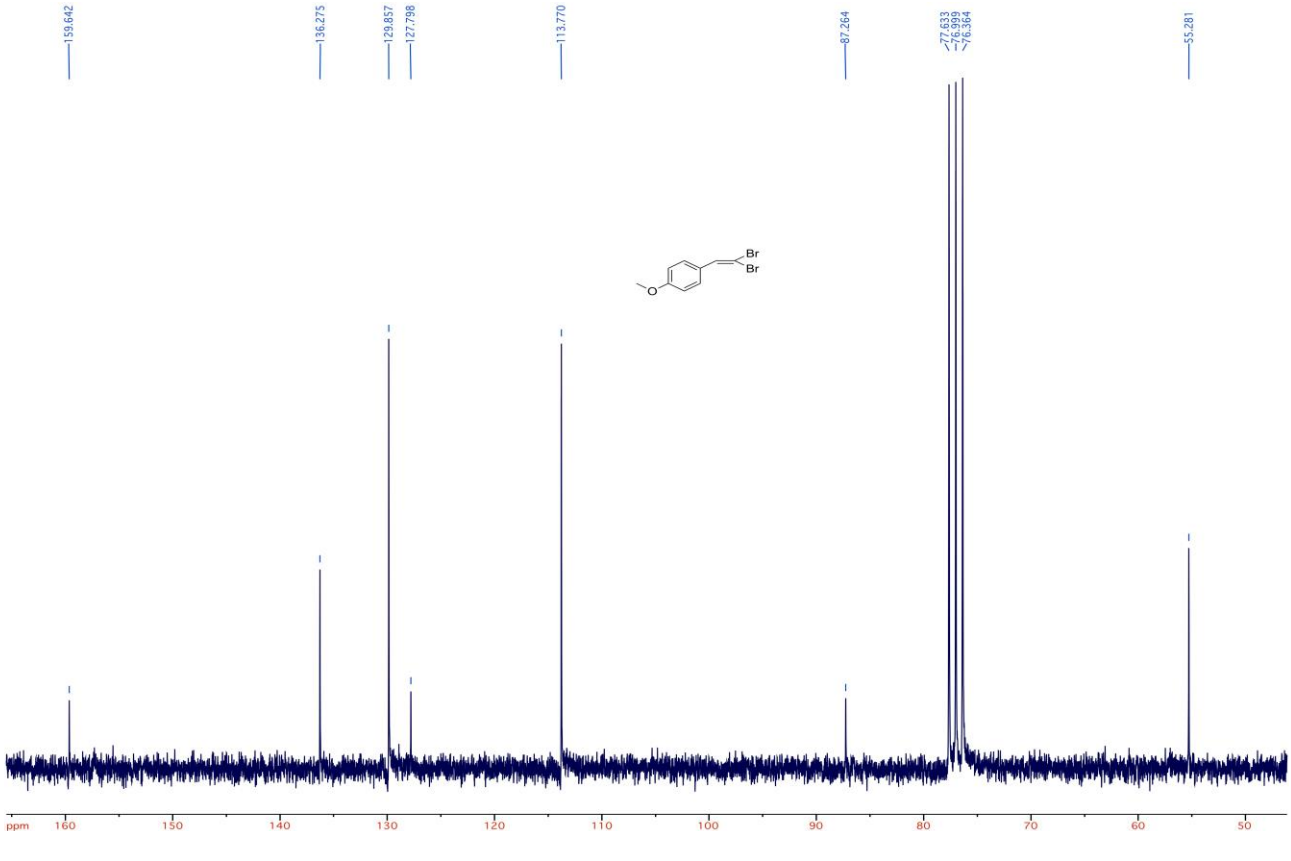

159.642
136.275
129.857
127.798
113.770
87.264
77.633
76.999
76.364
55.281
Br
Br
O
ppm
160
150
140
130
120
110
100
90
80
70
60
50

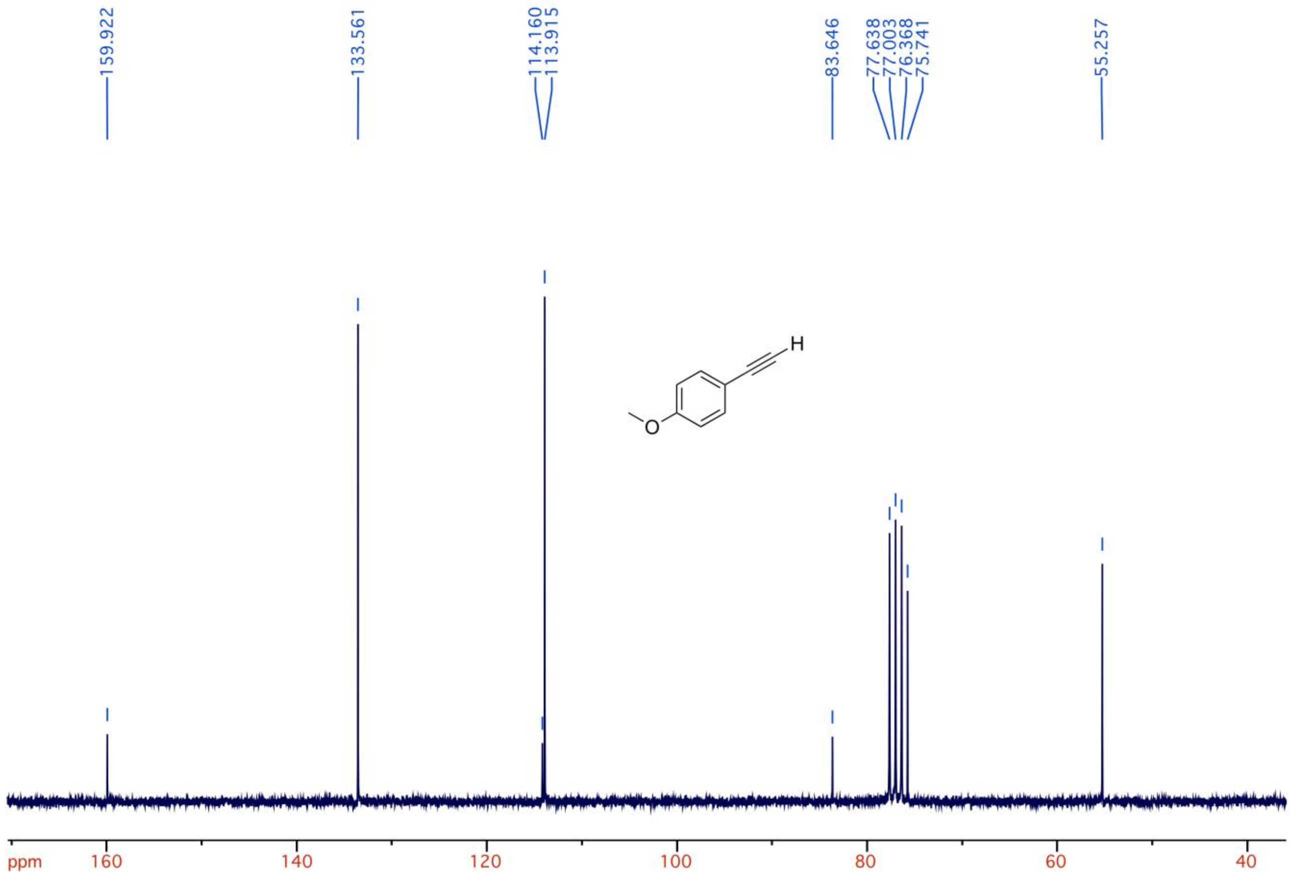
159.922
133.561
114.160
113.915
83.646
77.638
77.003
76.368
75.741
55.257
H
O
ppm
160
140
120
100
80
60
40

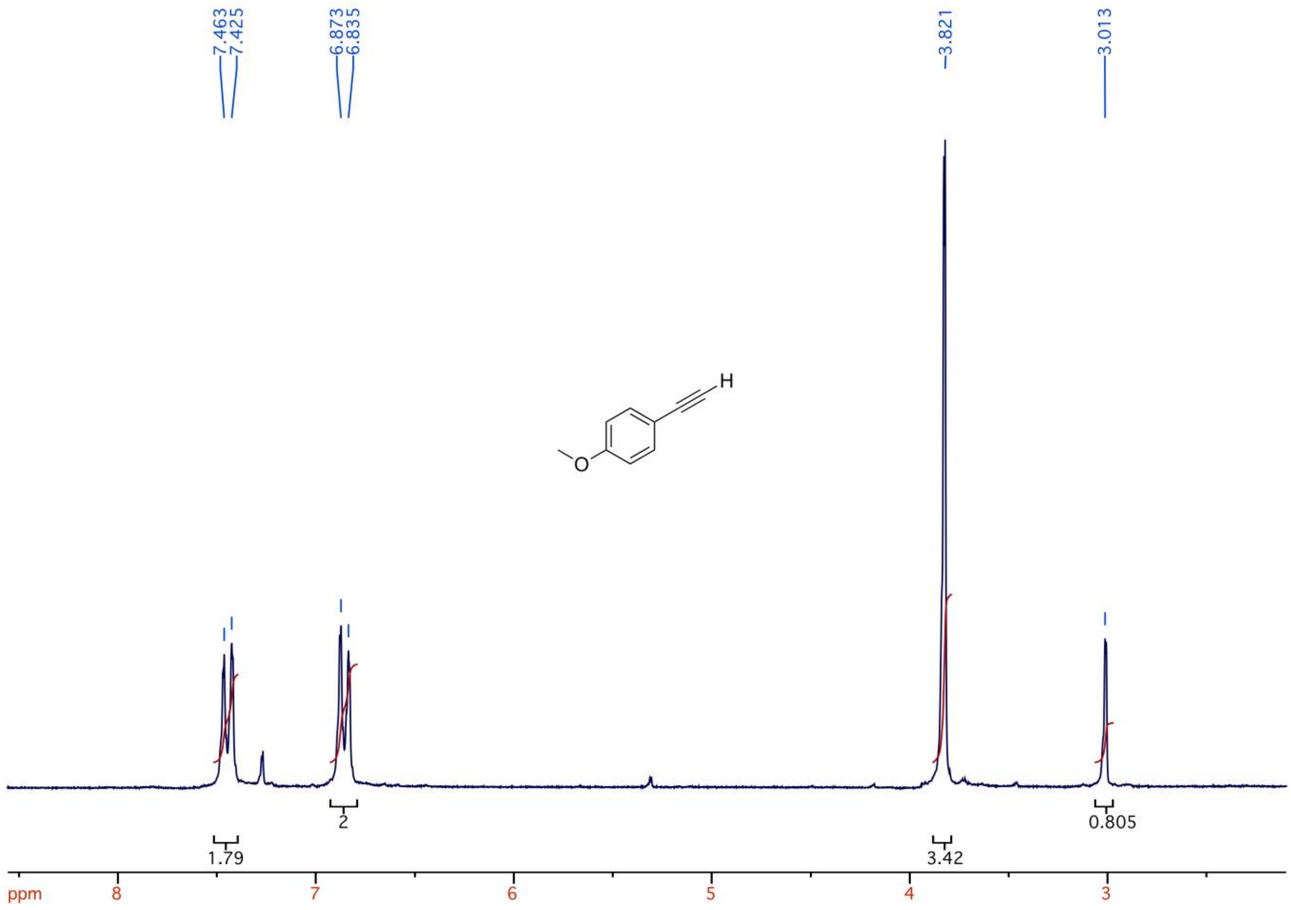
7.463
7.425
6.873
6.835
3.821
3.013
H
O
1.79
2
3.42
0.805
ppm
8
7
6
5
4
3

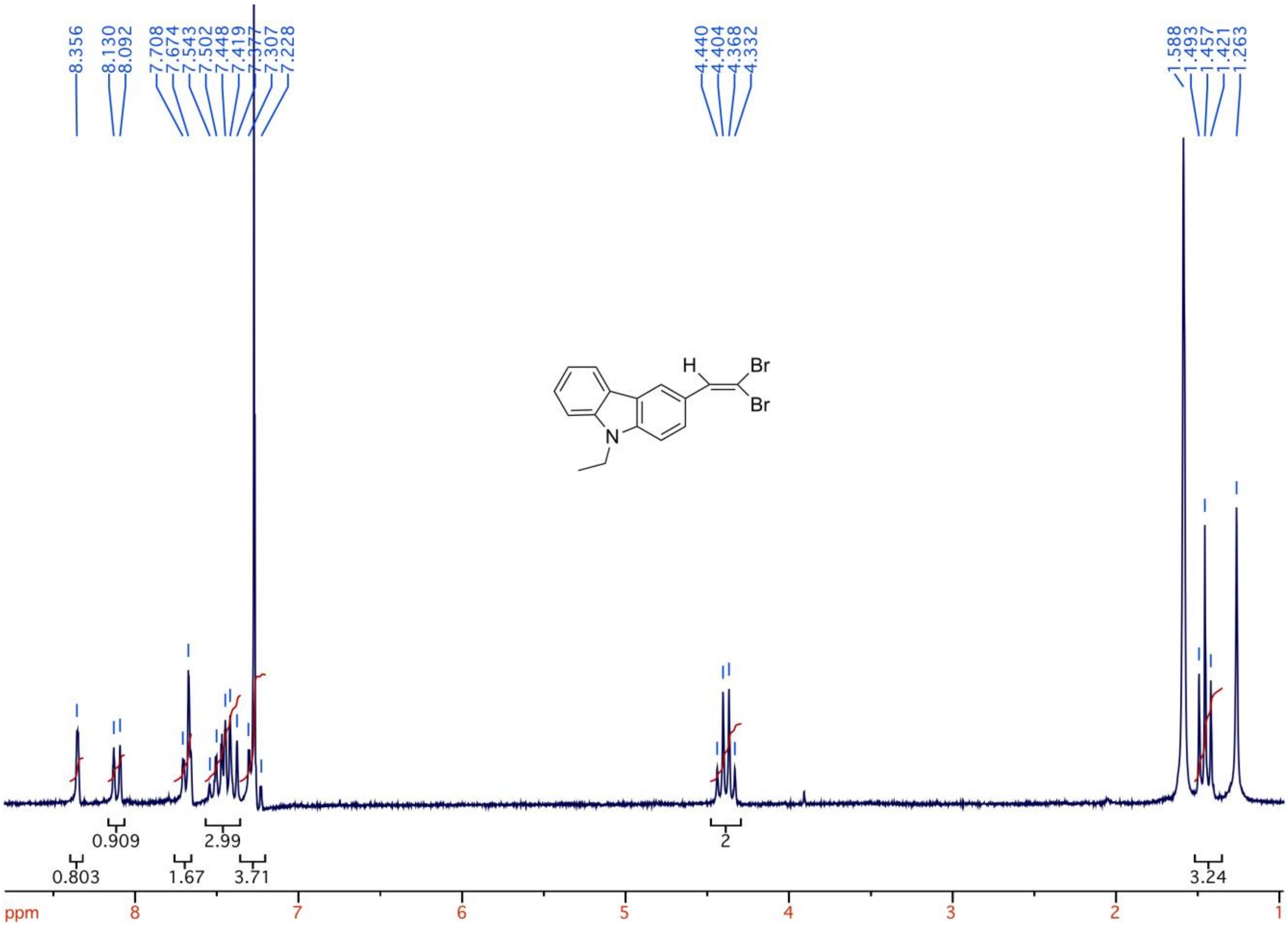
8.356
8.130
8.092
7.708
7.674
7.543
7.502
7.448
7.419
7.377
7.307
7.228
4.440
4.404
4.368
4.332
1.588
1.493
1.457
1.421
1.263
H
Br
Br
N
0.909
2.99
2
0.803
1.67
3.71
3.24
ppm
8
7
6
5
4
3
2
1

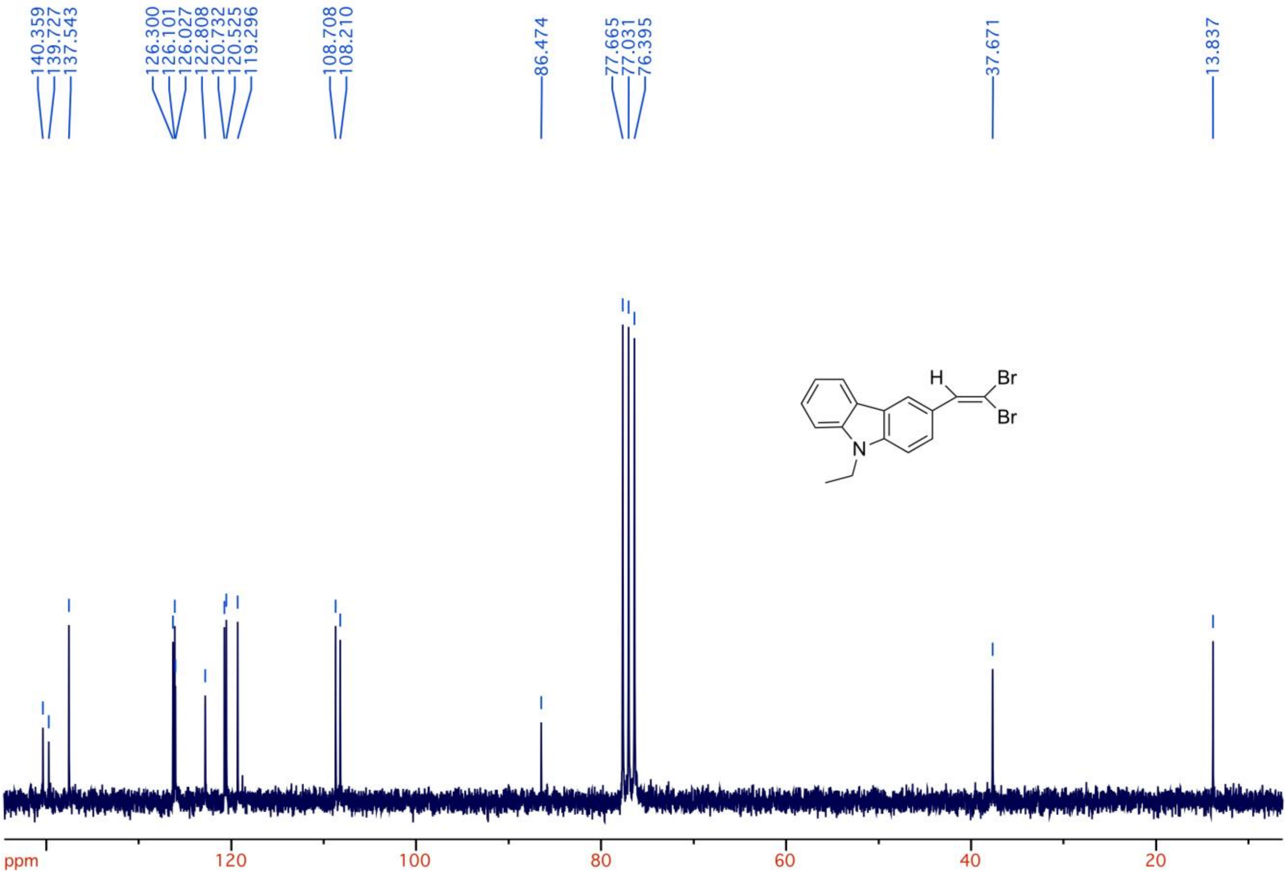
140.359
139.727
137.543
126.300
126.101
126.027
122.808
120.732
120.525
119.296
108.708
108.210
86.474
77.665
77.031
76.395
37.671
13.837
H
Br
Br
N
ppm
120
100
80
60
40
20

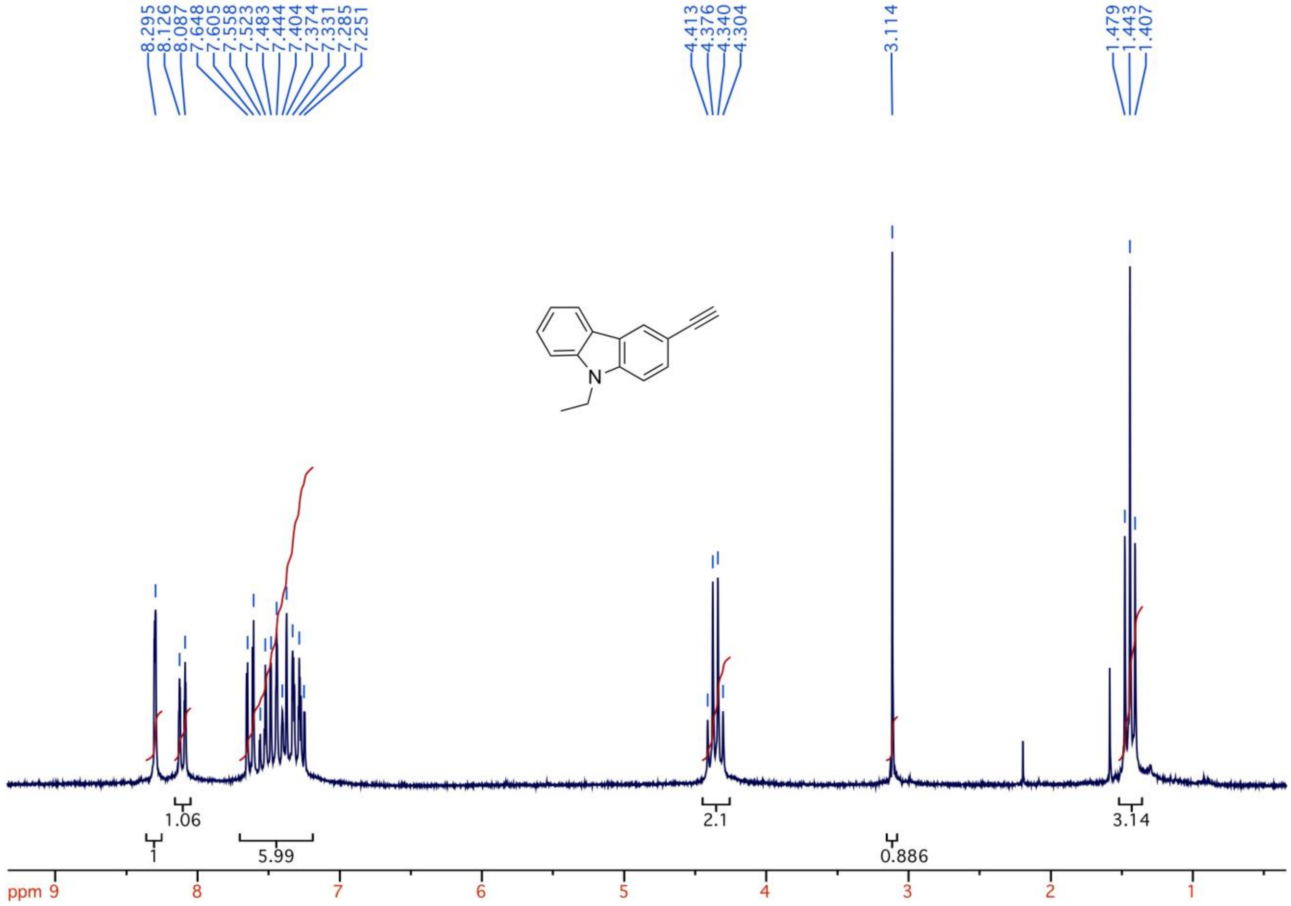
8.295
8.126
8.087
7.648
7.605
7.558
7.523
7.483
7.444
7.404
7.374
7.331
7.285
7.251
4.413
4.376
4.340
4.304
3.114
1.479
1.443
1.407
N
1.06
2.1
3.14
1
5.99
0.886
ppm 9 8 7 6 5 4 3 2 1

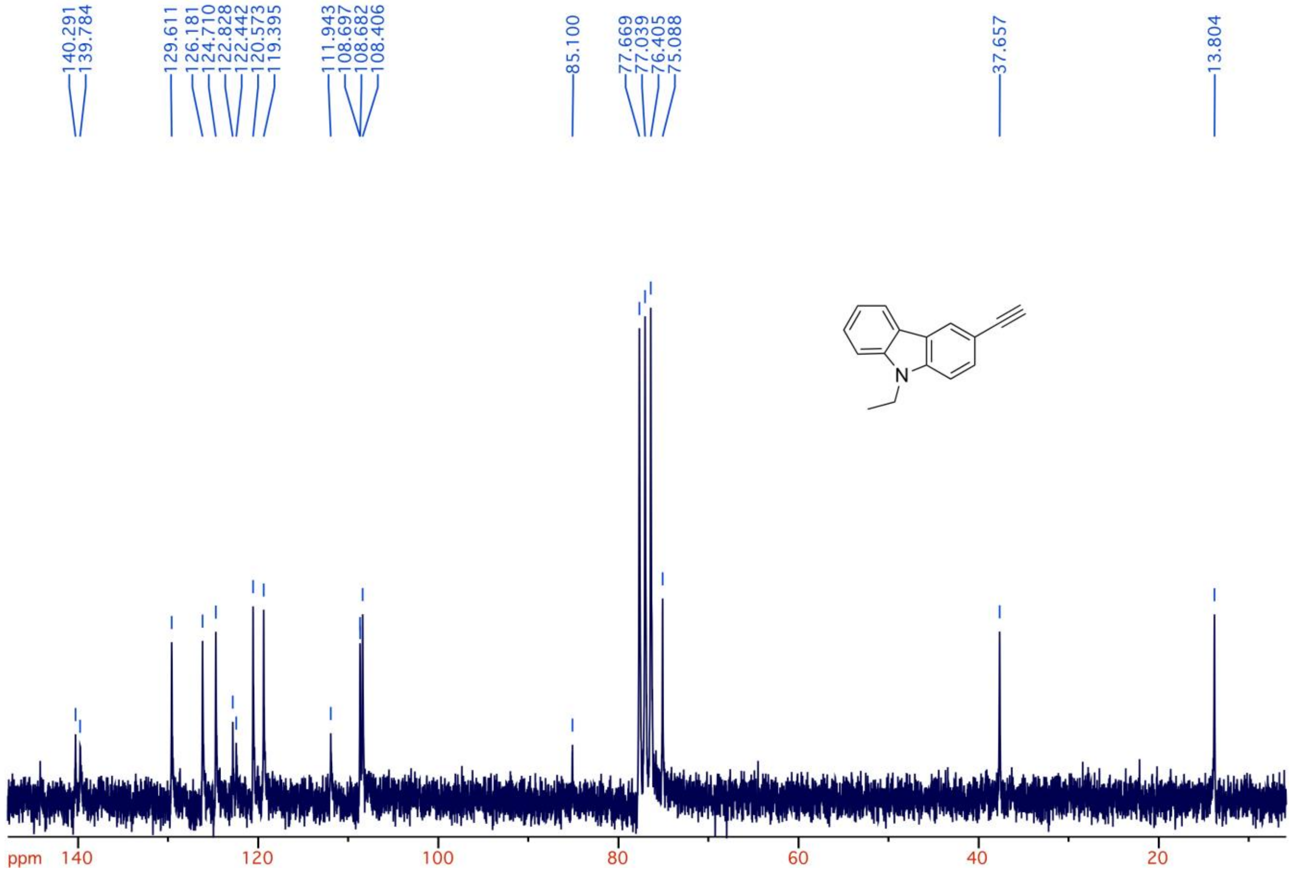
140.291
139.784
129.611
126.181
124.710
122.828
122.442
120.573
119.395
111.943
108.697
108.682
108.406
85.100
77.669
77.039
76.405
75.088
37.657
13.804
N
ppm 140 120 100 80 60 40 20
